# Genome-Wide Identification of *Pseudomonas aeruginosa* Virulence-Related Genes Using a *Caenorhabditis elegans* Infection Model

**DOI:** 10.1371/journal.ppat.1002813

**Published:** 2012-07-26

**Authors:** Rhonda L. Feinbaum, Jonathan M. Urbach, Nicole T. Liberati, Slavica Djonovic, Allison Adonizio, Anne-Ruxandra Carvunis, Frederick M. Ausubel

**Affiliations:** 1 Department of Genetics, Harvard Medical School, and Department of Molecular Biology, Massachusetts General Hospital, Boston, Massachusetts, United States of America; 2 Center for Cancer Systems Biology (CCSB) and Department of Cancer Biology, Dana-Farber Cancer Institute, and Department of Genetics, Harvard Medical School, Boston, Massachusetts, United States of America; The University of North Carolina at Chapel Hill, United States of America

## Abstract

*Pseudomonas aeruginosa* strain PA14 is an opportunistic human pathogen capable of infecting a wide range of organisms including the nematode *Caenorhabditis elegans*. We used a non-redundant transposon mutant library consisting of 5,850 clones corresponding to 75% of the total and approximately 80% of the non-essential PA14 ORFs to carry out a genome-wide screen for attenuation of PA14 virulence in *C. elegans*. We defined a functionally diverse 180 mutant set (representing 170 unique genes) necessary for normal levels of virulence that included both known and novel virulence factors. Seven previously uncharacterized virulence genes (ABC transporters PchH and PchI, aminopeptidase PepP, ATPase/molecular chaperone ClpA, cold shock domain protein PA0456, putative enoyl-CoA hydratase/isomerase PA0745, and putative transcriptional regulator PA14_27700) were characterized with respect to pigment production and motility and all but one of these mutants exhibited pleiotropic defects in addition to their avirulent phenotype. We examined the collection of genes required for normal levels of PA14 virulence with respect to occurrence in *P. aeruginosa* strain-specific genomic regions, location on putative and known genomic islands, and phylogenetic distribution across prokaryotes. Genes predominantly contributing to virulence in *C. elegans* showed neither a bias for strain-specific regions of the *P. aeruginosa* genome nor for putatively horizontally transferred genomic islands. Instead, within the collection of virulence-related PA14 genes, there was an overrepresentation of genes with a broad phylogenetic distribution that also occur with high frequency in many prokaryotic clades, suggesting that in aggregate the genes required for PA14 virulence in *C. elegans* are biased towards evolutionarily conserved genes.

## Author Summary

*Pseudomonas aeruginosa* is an opportunistic human pathogen that can also infect a wide range of model organisms, including the nematode *Caenorhabditis elegans*. To identify *P. aeruginosa* genes that play key roles in the pathogenic process, we performed a screen for mutants that exhibited reduced ability to kill *C. elegans* using a previously constructed non-redundant library representing approximately 80% of the non-essential *P. aeruginosa* PA14 genes. We defined a functionally diverse set of 180 *P. aeruginosa* mutants (representing 170 unique genes) necessary for normal levels of virulence that included both known and novel virulence factors. The major contributors to *P. aeruginosa* virulence in the *C. elegans* infection model were not secretion systems or their corresponding effectors, but rather regulators (particularly ones that are involved in quorum sensing) and genes likely to play key roles in survival of *P. aeruginosa* within the host intestine. Moreover, these putative *P. aeruginosa* virulence genes are neither overrepresented in strain-specific regions nor in horizontally acquired genomic islands and furthermore tend to have orthologs that are widely distributed across sequenced prokaryotic species. These data underscore the diversity of pathways involved in virulence, and especially the importance of highly conserved genes for *P. aeruginosa* virulence in the *C. elegans* host model.

## Introduction

*Pseudomonas aeruginosa*, an opportunistic Gram-negative human pathogen, is one of the leading causes of hospital-acquired infections. In the context of a breakdown in host defenses, it is capable of infecting a plethora of tissue types, causing both acute and chronic infections. Burn victims as well as immunocompromised, mechanically ventilated, and cystic fibrosis (CF) patients are particularly susceptible to *P. aeruginosa* infection [1]. Over the last few decades, a steady increase in drug resistant *P. aeruginosa* strains has made antibiotic treatment more difficult [2]. In part because no new antibiotics effective against *P. aeruginosa* are imminently available as treatment options, the pressing need for drugs to fight this pathogen has focused study on its virulence factors as potential drug targets, and more generally energized a search for novel anti-infectives [3]–[5].

One likely reason that *P. aeruginosa* is a common nosocomial pathogen is because it is capable of thriving in a wide variety of environmental niches, including surfaces in hospital rooms, water, soil and plants [6]. Consistent with its ecological ubiquity, *P. aeruginosa* has a relatively large genome that presumably promotes survival in diverse habitats. In addition to inhabiting a wide variety of ecological niches, *P. aeruginosa* is also a multi-host pathogen, capable of infecting hosts as divergent as amoebae, plants, insects, flies, nematodes, and mice [7]–[13]. Progress in fighting *P. aeruginosa* infections will be aided by a fundamental understanding of the myriad ways that *P. aeruginosa* can survive in different environments and cause disease in diverse hosts.

Our laboratory has developed a *Pseudomonas aeruginosa* - *Caenorhabditis elegans* infection-based model for studying host-pathogen interactions that is genetically tractable from both the perspectives of the host and the pathogen. This model (referred to as “slow-killing” or SK), which primarily utilizes *P. aeruginosa* strain PA14 [8], requires live bacteria and a set of bacterial virulence factors that distinguish it from rapid toxin-mediated PA14 killing of *C. elegans* (“fast killing” or FK) that occurs on high osmolarity media [12], [14]. Under standard laboratory conditions [15], *C. elegans* animals have a normal lifespan of approximately two weeks when feeding on non-pathogenic *Escherichia coli* strain OP50, and OP50 does not accumulate in the *C. elegans* intestine during the first few days of life. In contrast, when *C. elegans* are transferred at the L4 larval stage from *E. coli* OP50 to a lawn of *P. aeruginosa* strain PA14, the animals die in two-three days [12]. A few PA14 cells initially accumulate in the anterior and posterior portions of the nematode intestine, then over the course of one to two days bacteria spread throughout the intestine and the intestinal lumen becomes severely distended with a corresponding reduction in volume of the intestinal epithelial cells. Ultimately, PA14 cells move across the intestinal epithelial barrier destroying tissue, but it is not known whether tissue invasion is required for killing [16].

*C. elegans* rapidly responds to the presence of pathogenic PA14 by enhancing the transcription of hundreds of genes including a number of predicted secreted proteins (C-type lectins, CUB-domain containing proteins, ShK toxins) that may have antimicrobial or detoxifying activity [17], [18]. Two of the major classes of PA14 response genes, C-type lectins and CUB-like domain containing proteins, also play a role in mammalian innate immunity [19]–[21]. In *C. elegans*, many immune response genes are regulated by the PMK-1 p38 mitogen-activated protein kinase (MAPK), the terminal MAPK in an evolutionary conserved signaling cassette required for defense against pathogens in both nematodes and mammals. Approximately 25% of the *C. elegans* genes regulated by PMK-1 are also induced in response to *P. aeruginosa* PA14 [18] and *C. elegans* p38 MAPK pathway mutants exhibit enhanced sensitivity to PA14 as well as a variety of other bacterial and fungal pathogens [22]–[24].

Several hundred genes have been implicated in *P. aeruginosa* virulence based on data obtained from a wide variety of host infection models (Pseudomonas.com). Many of the well-studied *P. aeruginosa* virulence-related factors participate directly or indirectly in physical interactions with the host cell and/or host proteins, including secretion systems (type II, type III, type VI) and associated effectors (including ExoT, ExoU, ExoS, ToxA, phospholipase C, and alkaline protease), flagella, and structures involved in attachment to host cells such as type IV pili. Other recognized virulence factors include those involved in quorum sensing (AHL and PQS systems), iron acquisition (pyochelin, pyoverdine), small molecule/toxin synthesis (phenazines, hydrogen cyanide), alginate, LPS, and biofilm. Not all of these classes of virulence-related factors play a significant role in *P. aeruginosa* strain PA14 virulence in *C. elegans*; for example, the Type III secretion system and its associated virulence effectors have been shown to play no detectable role in nematode killing, in contrast to playing key roles in pathogenesis in mammals and insects [25]. However, a variety of *P. aeruginosa* PA14 virulence factors required for killing *C. elegans* in the SK infection model are also required for full pathogenesis in mammalian models, including the quorum sensing regulators LasR and RhlR, the two component regulator GacA, the alternate sigma factor RpoN, the periplasmic protease MucD, and the phosphoenolpyruvate-protein phosphotransferase and transcriptional regulator PtsP [13], [26]–[29]. Additionally *P. aeruginosa* virulence-related factors involved in LPS biogenesis and type IV pilus assembly and function also play a role in both mammalian and *C. elegans* hosts [30]–[33].

A common theme that has emerged from the study of bacterial virulence in a wide variety of pathogens and hosts is an association linking virulence-related genes with regions of genomic plasticity, including genomic pathogenicity islands (PAIs) [34], so-called “replacement islands” harboring the pyoverdine [35] and O-antigen biosynthetic loci [36], and plasmids [37]. These findings indicate that horizontal gene transfer has played an important role in the evolution of virulence. For example, phylogenetic analysis of three sub-families of the type III effector HopZ in the plant pathogen *Pseudomonas syringae*, suggested that at least two were acquired by *P. syringae* from disparate donors [38]. Analysis of the occurrence of virulence factors across many pathogen genomes has suggested that there is an overrepresentation of virulence factors on genomic islands [39], and two virulence factor- containing pathogenicity islands, PAPI-I and PAPI-II, have been identified in *P. aeruginosa* [40].

Although there are many published examples linking virulence-related factors to putative pathogenicity islands, a preliminary study from our laboratory showed that the presence of genes occurring in the highly virulent strain PA14, but not in the less virulent strain PAO1, could not be correlated with increased virulence across a wider sampling of strains, suggesting that virulence is a combinatorial and multifactorial product of the interactions of many potential virulence factors [32]. These data were seemingly at odds with the expected over-representation of virulence factors in strain-specific regions such as genomic islands, but were not definitive because only a limited set of virulence factors were available for analysis. Further, comparison of the sequences of five pathogenic *P. aeruginosa* strains suggested that virulence was primarily encoded by a core *P. aeruginosa* genome [41], a set of genes shared by all strains, and not the auxiliary genome defined by regions of genomic plasticity that are strain-specific. An unbiased comprehensive list of *P. aeruginosa* virulence factors required to cause disease in *C. elegans* would allow us to better understand what genes are the major contributors to virulence and whether these genes are primarily located in regions of genome plasticity or not. We considered this question worthy of investigation because it seemed likely to us that the virulence factors of an opportunistic multi-host pathogen might as a group be distinct from the virulence factors of host-specific pathogens.

We report here the results of a genome-wide screen using a previously constructed non-redundant PA14 transposon library consisting of 5850 members that represents insertions in approximately 80% of the non-essential ORFs in *P. aeruginosa* strain PA14 [42]. Previous studies to identify *P. aeruginosa* virulence factors *in vivo* using a number of different technologies and infection models have been limited by the complexity and redundancy of mutant collections or screening procedures [13], [26], [27], [43]–[51]. We examined the genes identified in this genome-wide screen for their functions, presence on putative and characterized genomic islands, and their phylogenetic distribution across prokaryotes. We demonstrate that the major genes contributing to PA14 virulence in *C. elegans* are not enriched on genomic islands, are not PA14 or *P. aeruginosa* specific genes, and may in fact be biased for ancient genes common to many other prokaryotic species.

## Results

### Genome-Wide Screen for PA14 Mutants Attenuated in *C. elegans* Killing

#### Primary screen

**Figure 1:**
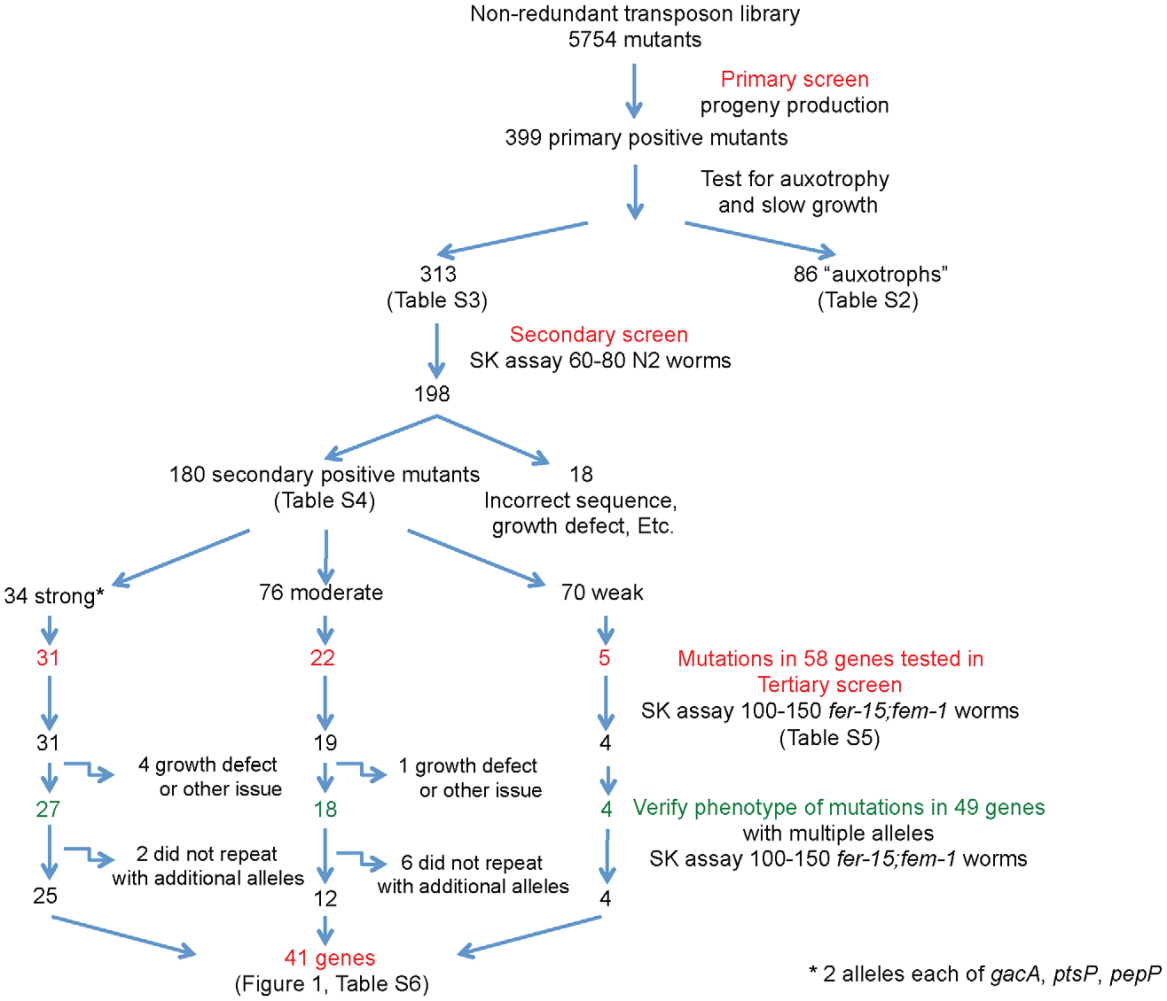
Pipeline of screen for PA14 virulence-attenuated mutants in *C. elegans*. The three screening steps for identification of *P. aeruginosa* PA14 virulence-attenuated mutants are outlined; details of the screens are presented in the Materials and Methods and the text. The number of mutants obtained after each round of screening, as well as those removed from the pool for various reasons, is shown. Note that the 313 mutants identified in the primary screen and the 180 from the secondary screen represent 294 and 170 unique genes respectively because some genes were represented by multiple mutants, and a small fraction of mutants were in intergenic regions (see text). In the tertiary screen a single mutant defined each gene.

A non-redundant (NR) library of *MAR2xT7* transposon insertion mutants representing approximately 75% of the total and 80% of the non-essential ORFs in *P. aeruginosa* PA14 was screened for attenuation of virulence in an infection-based model of *C. elegans* killing (see Figure 1, Figure S1 and Table S1 for an overall scheme of the screen). In this infection assay, called “slow killing” (SK), nematode death requires live bacteria and correlates with accumulation of bacteria in the nematode intestine [12], [16]. Ideally, we would have screened the entire non-redundant transposon library using the SK assay, but the relatively large number of mutants (5850) in the library made direct quantitation of killing kinetics impractical for a primary screen. In previous work [13] and in new experiments with known virulence-attenuated mutants (Figure S2), we observed that the quantity and maturity of the brood size of an infected hermaphroditic worm is generally inversely correlated with the virulence of the infecting PA14 strain. Under our standard assay conditions, wild-type PA14 kills *C. elegans* rapidly enough to dramatically diminish the number of progeny produced, and the few larvae that hatch do not reach maturity. However, PA14 mutants with reduced virulence (such as *gacA, lasR, mucD*; Figure S2) allow the *C. elegans* hermaphrodites to produce a significant brood that is able to reach adulthood and in turn become gravid, often consuming the entire bacterial lawn. Based on these data, we concluded that we could use PA14-mediated nematode brood size reduction, a much less time consuming assay than monitoring death of a population of worms, as a proxy for PA14-mediated killing.

A total of 5,754 mutants (the complete 5,850 PA14 NR library minus one 96-well plate consisting of previously characterized slow-growing mutants) was screened twice on SK agar in standard 6-well assay plates for mutants that permitted an increase in *C. elegans* progeny number or allowed the brood to mature to adulthood when compared to wild-type PA14. In this primary screen, 399 mutants (corresponding to 368 genes and three mutants in intergenic regions) were identified as potentially attenuated in virulence (Figure 1, Figure S1 and Table S1). From the set of 399 putative virulence-attenuated mutants, 86 auxotrophic or growth-defective mutants corresponding to 74 genes (Table S2) were identified by replica plating on minimal medium. Some but not all of the 86 auxotrophic mutants formed noticeably thin lawns on the SK agar plates. We reasoned that the 86 mutants with fundamental growth defects were not relevant to our study of virulence and we eliminated them from future studies. This left 313 putative virulence-related mutants corresponding to 294 distinct genes and three mutants in intergenic regions (Table S3).

#### Secondary screen

The 313 putative PA14 virulence-related mutants that did not appear to be dependent on the addition of amino acids or nucleotides for growth on minimal media were re-tested using *C. elegans* slow killing assays. Specifically, the survival of 60–80 wild-type N2 Bristol worms was quantified at two or three different time points following manual transfer of individual worms from *E. coli* OP50 to a lawn of each PA14 mutant. In addition, the number of progeny produced was scored after four days. Each batch of mutants tested in the secondary screen included two known attenuated mutants, the strongly avirulent quorum sensing regulatory mutant *lasR* and the moderately attenuated type IV pilus protein mutant *pilA*. The mutants from the secondary screen were ranked with respect to these controls. About 63% (198) of the 313 non-auxotrophic mutants from the primary screen exhibited an attenuated killing phenotype or increased brood size in the secondary screen (Figure 1, Table S1). The insertion sites of *MAR2xT7* in the attenuated mutants from the secondary screen were re-sequenced to verify their identity and the mutants were rescored for readily apparent growth defects in overnight cultures or on plates. As a result, 18 mutants that had either incorrectly annotated sequence or were slow growing were removed from the list. Of the 180 remaining virulence-attenuated mutants from the secondary screen (representing 170 genes and one mutant in an intergenic region), 34 were strongly attenuated (similar to *lasR*), 76 were moderately attenuated (greater than or equal to *pilA*), and 70 were weakly attenuated (less than *pilA* but allowing greater parental or progeny survival than wild-type PA14) (Table S4).

Previously in our laboratory, 17 PA14 mutants were shown to exhibit an attenuated phenotype in a standard *C. elegans* SK assay. Of these 17, 15 were identified in relatively small-scale forward genetic screens (*aefA, lasR, mtrR, ptsP, gacA, gacS*, PA14_03370, ORF_10 (PA14_23420), ORF_11 (PA14_23430), PA14_27680 (GID6172), PA14_27700 (GID6170), *pilC*, PA14_59010, PA14_59070 and *pilW*) and two by a candidate gene approach testing predicted virulence factors (*rpoN, mucD*). Of these 17 genes, 16 are represented by mutants in the PA14 non-redundant library (*lasR* is absent). One of these 16, *rpoN*, grows very slowly under our conditions and was not assayed. Thus 15 previously identified mutants could potentially have been recovered in the screen of the non-redundant library. In fact, nine of these 15 previously identified mutants were identified in the primary screen and eight of these nine also scored as positives in the secondary screen (strongly attenuated *ptsP, gacA, gacS*, and PA14_27700 (GID6170), moderately attenuated i.e. close to the attenuation of *pilA*, ORF_11, *mucD*, and PA14_27680 (GID6172), and very weakly attenuated PA14_03370). At least four of the remaining seven previously identified virulence-related genes (ORF_10, *pilC*, PA14_59010, PA14_59070) that we did not isolate in the secondary screen exhibited virulence-related phenotypes approximately equal to *pilA*, which has a phenotype at the lower limit of sensitivity for recovery in the progeny-based screen (note an ORF-10 mutant was recovered in the primary screen but not the secondary). Although *pilC* and ORF_10 were not recovered in the secondary screen, other type IV pilus and O-antigen synthesis mutants (*pilF, pilW, pilU* and ORF_11) were identified. Based on these data, we conclude that the genome-wide screen that we carried out to identify PA14 virulence-related factors was successful in identifying the majority of the virulence-related mutants in the non-redundant library with strong killing-attenuated phenotypes, but that many potential mutants with relatively weak attenuated phenotypes were probably overlooked.

We compared the genes identified in our screen to a set of 241 *P. aeruginosa* strain PA14 virulence-related genes downloaded from the Virulence Factor Database (VFDB). VFDB is, a compilation of virulence factors from a wide variety of pathogens in numerous host systems that includes virulence factor sets for PA14 and for three other *P. aeruginosa* isolates [52] (http://www.mgc.ac.cn/VFs/). The VFDB set for PA14 incorporates well-studied virulence factor classes, most abundantly those involved in adherence/motility (flagella, type IV pilus, LPS), alginate, rhamnolipids, iron uptake (pyochelin and pyoverdine), quorum sensing, global regulators (GacA/GacS), proteases (alkaline protease, LasA, LasB), lipases (PlcH, PlcN), secretion systems and associated effectors (type III, type VI), pyocyanin pigment and toxins (ToxA, hydrogen cyanide). The degree of overlap between the VFDB genes and those identified in our screen increased between the primary and secondary round of screening, with VFDB genes constituting 10.2% (30 of 294) of the genes identified in the primary screen and 11.8% (20 of 170) of the genes identified in the secondary screen (Figure S3).

#### The collection of avirulent secondary positive hits does not exhibit a strong functional bias

The 170 genes (represented by 180 mutants) from the secondary screen were grouped by the 27 functional protein classes used in the annotation of the PA14 genome (http://ausubellab.mgh.harvard.edu/cgi-bin/pa14/annotation/statistics.cgi) and the fraction of genes in each functional class was compared to that of the total PA14 genome (Table S4). Only one functional class, LPS and capsule biosynthesis, was marginally statistically overrepresented (p-value = 0.013) after FDR correction. In addition, the 170 genes were mapped onto the KEGG pathway database to determine whether particular biochemical pathways were enriched and were categorized by GO term with DAVID. Neither the KEGG pathway nor the GO term analysis revealed any significant overrepresentation of pathways or GO terms.

#### The PA14 secondary virulence screen did not enrich for known secretion systems or secreted effectors

Canonical virulence factors such as those present in VFDB are enriched in extracellular proteins (10% in VFDB vs. a little over 1% in the PA14 genome and NR mutant set) and in various secretion systems (22% in VFDB vs. approximately 2% in the PA14 genome). In contrast, mutations with consistent or strong phenotypes in any of the known secretion systems or effectors were significantly underrepresented in our genome-wide screen. Among 62 documented PA14 secreted proteins and their chaperones [53] (PA14 database at http://ausubellab.mgh.harvard.edu/cgi-bin/pa14/annotation/start.cgi), 55 of which correspond to mutants in the PA14 NR Set, only one was also found in the 180 virulence-attenuated mutant set from the secondary screen. Similarly, although our primary screen identified 15 of 97 NR set mutants annotated to be secretion apparatus proteins or their chaperones (type II, III, V and VI), only six of these were retained after the secondary screen. Three of the secretion apparatus loci mutants isolated in the secondary screen (type II secretion loci *xcpT*, *xcpZ* and *secB*) exhibited only slight attenuation of virulence and three had moderate attenuation of virulence roughly equivalent to the *pilA* mutant control (typeII *tatC* and type VI HSI-I locus clpVI and type VI HIS-II *fha2*). Although we isolated two type VI mutants in our screen it is unclear what this means as many of the type VI structural loci were not identified, and two large deletions of type VI HSI-II and HIS-III had little effect on virulence (data not shown). These data do not strongly implicate a particular secretion system as predominant in PA14 virulence in *C. elegans* and are consistent with previous work from our lab, which showed that the type III secretion system mutants had no detectable attenuation of virulence in *C. elegans* [25]. On the other hand, the failure to observe a phenotype with the secretion defective mutants may be a consequence of built-in redundancy.

#### Comparison of the 170 *P. aeruginosa* putative virulence genes identified in *C. elegans* with genes required for virulence in a rat chronic infection model

In order to evaluate the degree to which virulence genes from our screen represent putative conserved virulence determinants, we compared the 170 genes identified in our secondary screen with the largest available set of genes identified in another unbiased screen, a negative signature tagged mutagenesis (STM) selection for *P. aeruginosa* mutants defective in virulence in a rat chronic respiratory infection model [46]. It is notable that like the 170 genes identified in our screen, and in contrast with the VFDB set, the 148 *P. aeruginosa* genes identified by Potvin et al (2003) by STM also appear as a group to possess a broad distribution across all functional classes. The *P. aeruginosa* mutant set identified in the rat chronic infection model exhibits an underrepresentation of secreted proteins and secretion systems and includes a number of auxotrophic mutants and many mutants in genes with enzymatic functions not previously linked to pathogenesis, reminiscent of the mutants identified in *C. elegans*. Only five genes identified in the rat infection model were also found in the 170 gene set from our secondary screen and only one of these was also found in the VFDB (Figure S4). A number of well-studied *P. aeruginosa* virulence factors, required for slow killing in *C. elegans* and in some mammalian infection models, including *gacA*, *lasR*, *rhlR*, *ptsP*, *mucD*, and *rpoN*, are absent from the rat chronic infection set. We do not know if this reflects a difference between chronic and acute infections, nor whether the *C. elegans* model is more analogous to acute or chronic infection in mammals. These data suggest that genes required for virulence under a particular set of circumstances are highly dependent on host model, infection site, and most likely phase of infection.

#### Tertiary screen

The relatively large number of positive mutants (180 corresponding to 170 genes) from the secondary screen made it necessary to prioritize mutants for further characterization. A subset of 58 mutants/genes was selected based on strength of attenuation, annotation (biased toward regulators and mutants with functions previously implicated in virulence), whether they were in putative operons or functionally grouped with other attenuated mutants, and whether they exhibited normal doubling times (see Materials and Methods). These mutants (Table S5) included 31 mutants with strongly attenuated virulence similar to that of the *lasR* quorum sensing mutant (34 such mutants were isolated but *gacA*, *ptsP* and *pepP* were represented by two mutants each and a single allele of these three genes was carried through the tertiary screen), 22 mutants with moderately attenuated virulence (less attenuated than *lasR* but equal to or more attenuated than *pilA*), and five weakly attenuated mutants (less attenuated than *pilA*).

In the tertiary screen, each of the 58 selected mutants was subjected to SK assays using 100–150 *fer-15(b26)II;fem-1(hc17)IV* worms and the time to 50% survival was determined. Sterile *fer-15;fem-1* worms were used in these assays to avoid production of a brood that would complicate the scoring of death of the parental worms. *fer-15;fem-1* animals, while slightly more resistant to PA14 than N2 worms, have previously been used to study the transcriptional response of *C. elegans* to PA14 [18]. Importantly, wild-type N2 worms and *fer-15;fem-1* worms exhibit comparable relative susceptibilities to a *gacA* mutant [18]. Four mutants (PA4296, *rcsC*, *bkdA1*, and *norB*) did not re-test as avirulent in the tertiary assay, three (in ORFs PA2658, PA14_4560 and PA4745) exhibited impaired growth in LB overnight cultures that had been previously overlooked and two mutants were removed based on ambiguity as to which ORF they were inactivating (mutants #38436 and #5691) (Table S5). Thus, the tertiary screen substantiated the avirulent phenotype of 49 of the 58 mutants.

### Use of the Master Transposon Library to Verify Phenotypes and Examine the Genomic or Functional Context of Putative Virulence-Attenuated Mutants

#### Multiple transposon alleles confirm avirulent phenotypes

One of the bottlenecks in a large-scale screen such as the one described in this paper is verification of the mutant phenotypes. In general, the gold standard to correlate phenotype with genotype is to generate an in-frame deletion mutant and then complement the mutant in trans to establish that the phenotype observed is due to the defect in the identified gene. Because the goal of our screen was to obtain an overview of the major factors utilized by PA14 to infect and kill *C. elegans*, we were interested in characterizing a large collection of mutants. Generation of in-frame deletions for all the genes of interest was prohibitively time-consuming. As an alternative, we reasoned that by examining multiple independent *MAR2xT7* insertion mutations in candidate virulence-related genes, as well as insertions in adjacent genes within putative operons, we could: a) verify the avirulent phenotype of the original mutation, b) reduce the likelihood that the avirulent phenotype was due to polar effects of the original transposon insertion on downstream ORFs, and c) eliminate the possibility that second-site mutations were responsible for the less virulent phenotype. The NR PA14 library of 5,850 mutants was selected from more that 24,000 sequenced *MAR2xT7* transposon insertion mutations and on average each gene in the NR library is represented by 4.3 transposon insertions in this “master” library of 24,000 mutants [42].

**Figure 2:**
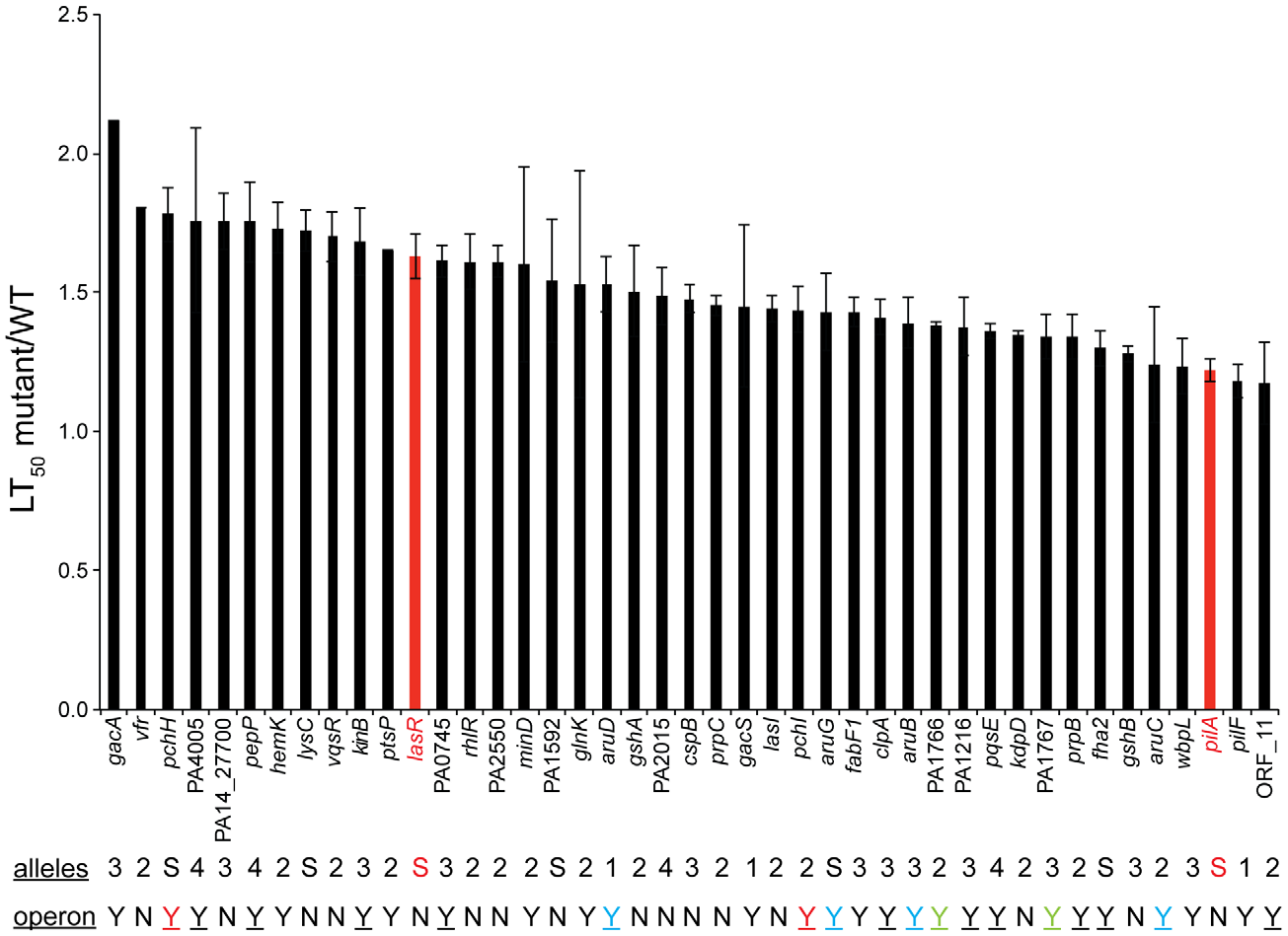
41 PA14 genes required for virulence in a *C. elegans* infection based killing model. The ratio of nematode survival on mutant PA14 to that on wild-type PA14 (mutant LT_50_/WT LT_50_) is presented for 41 mutants identified after three rounds of screening as well as for the known virulence-attenuated mutants, *lasR* and *pilA*. The time to 50% death (LT_50_) was calculated using a non-linear regression based on the Hill equation (Prism 5.0). 100–150 animals were tested in each experiment. Error bars represent the SEM of the ratios derived from at least two different experiments (lack of error bars indicates that the mutants for known virulence factors *gacA*, *ptsP* and *vfr* were tested only once). Red bars depict the ratio of the LT_50_ of *lasR* or *pilA* to WT PA14. The *lasR* and *pilA* mutants were generated previously (see Materials and Methods); there are no alleles of *lasR* or *pilA* in the NR library. The number of alleles tested with an avirulent phenotype is indicated by a number below the graph: 1 indicates that a single allele was tested but that there exist multiple alleles in the master transposon library, S indicates only a single allele was available in the library. Genes that are predicted to be in operons are indicated (Y = yes, N = no). Genes in a single operon are represented in the same color and an underline designates that other genes within the same operon were tested for their role in virulence.

**Figure 3:**
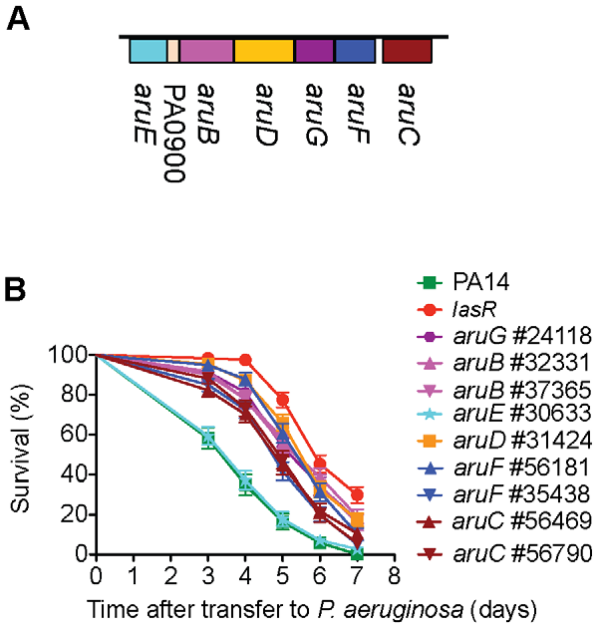
The catabolic arginine succinyltransferase (*aru*) operon is required for normal virulence in *C. elegans*. A) *aruFGDB* is transcribed as a unit; the transcriptional regulator *aruC* is transcribed separately. The *aruFGDB* operon encodes enzymes for the major aerobic route of arginine utilization as an energy, carbon and nitrogen source [56], [57]. In *P. aeruginosa* PA01, *aruE* belongs to a separate transcription unit [57]. B) *MAR2xT7* insertions in *aruC*, *aruF*, *aruG*, *aruD* and *aruB* all reduce the virulence of PA14. The single mutation in *aruE* has normal virulence.

**Figure 4:**
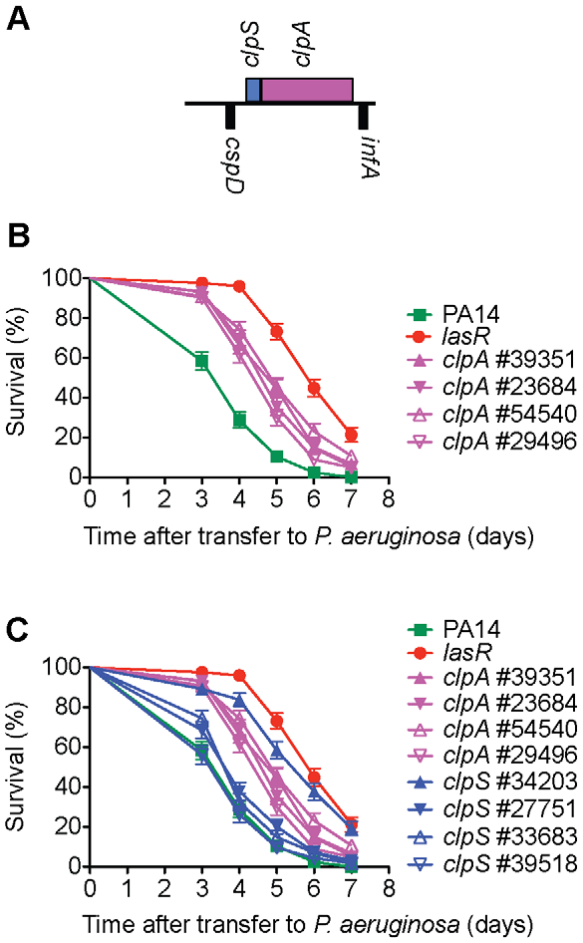
Multiple transposon alleles of *clpA*, but not *clpS*, are virulence-attenuated. ClpA is the chaperone subunit responsible for substrate recognition of the ClpAP ATP dependent protease common to Gram-negative proteobacteria [96]. A) *clpA* (PA2620) is the second gene of a two gene operon; it is preceded by *clpS* (PA2621), encoding a ClpAP adaptor protein that has been shown to bind to the N-terminus of ClpA and inhibit ClpAP degradation of some substrates while enhancing the degradation of others [97]. B) Four different *MAR2xT7* transposon insertion alleles of *clpA* are decreased in virulence in *C. elegans*. C) Three of four *MAR2xT7* insertion alleles of *clpS* exhibit wild-type levels of virulence. Only the *clpS* mutant (#34203) identified in the primary and secondary screens has a virulence-attenuated phenotype. A mutant in the ClpP proteolytic subunit (#52957) was also identified in our primary screen for virulence-attenuated mutants, but this mutant was defective in growth on minimal media and therefore was not analyzed further.

**Figure 5:**
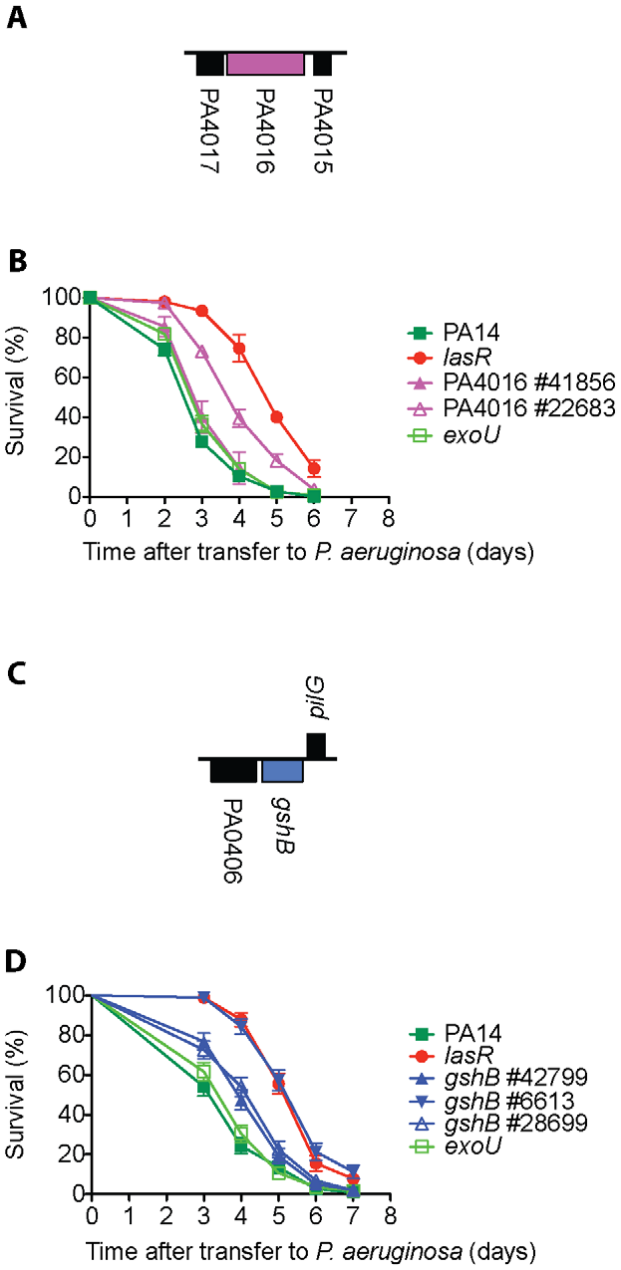
*ΔexoU* may be a sensitized background that can reveal virulence-associated genes. Deletion of the Type III effector protein ExoU has no statistically significant impact on PA14 virulence in *C. elegans* (B and D and [25]). A) Hypothetical protein PA4016 and adjacent loci PA4017 and PA4015; PA4016 is most likely a single gene transcription unit. B) A PA4016 *MAR2xT7* insertion mutant (#22683) in the *ΔexoU* strain background has attenuated virulence in *C. elegans*, but a second PA4016 insertion allele (#41856) in the WT strain does not. C) Glutathione synthetase, *gshB* (PA0407), is a single gene transcription unit. D) Multiple alleles of *gshB* exhibit reduced virulence in *C. elegans* but the *gshB* #6613 allele in the *ΔexoU* strain background is more attenuated.

Among the mutations in the 49 genes that re-tested as avirulent in the tertiary screen, there were 44 genes for which there was more than one corresponding transposon insertion. (However, three genes for which multiple insertion alleles existed, *gacS*, *pilF* and *aruD*, were only tested with single alleles. *gacS* and *pilF* had been previously implicated in virulence in *C. elegans* and in the case of *aruD*, the entire *aru* operon was shown to be virulence-attenuated (Figure 2 and Figure 3). A total of 72 additional insertion mutants corresponding to 41 of the 49 genes were tested in SK assays. These assays resulted in the elimination of eight genes (PA2089, PA0902, PA1032, PA0533, PA4016, *clpS*, *sltB1* and *pvdD*) from further consideration because the additional transposon insertions in these genes did not cause an attenuated killing phenotype (representative data are shown for *clpS* (Figure 4) and PA4016 (Figure 5B). In the case of *clpS*, for example, in contrast to the virulence-attenuated *clpS* NR allele mutant ID#34203 isolated in the screen (LT_50_ mutant/LT_50_ wild-type (WT) PA14 = 1.66), three additional independent alleles of *clpS* all exhibited killing kinetics similar to WT PA14 (LT_50_ mutant/LT_50_ WT PA14 = 1.11, 1.10 and 0.98 respectively) suggesting that the phenotype of mutant #34203 was aberrant.

#### *ΔexoU*, a potential sensitized genetic background

We noticed that in 4/8 cases where the phenotype of the mutant identified in the screen was not recapitulated by additional alleles (putative transcriptional regulator PA0533 mutant #22525, probable penicillin amidase PA1032 mutant #6114, hypothetical protein PA4016 mutant #22683, and *pvdD* PA2399 mutant #5205), the original allele was in a *ΔexoU* background, which carries an in-frame deletion of the type III effector ExoU [42]. The *ΔexoU* mutant behaved similarly to wild-type PA14 in nematode killing assays (Figure 5B, D and [25]). The observation that 50% of the mutants in which the original phenotype did not recapitulate with multiple alleles also contained a *ΔexoU* mutation despite the fact that less than 4% of the transposon insertions in the NR library are in the *ΔexoU* background suggests that the mutation in *exoU* might create a sensitized genetic background to identify other putative virulence factors. In support of this conclusion, two alleles of *gshB* (#42799 identified in our screen and #28669 from the master library) exhibited modest virulence attenuation (LT_50_ mutant/LT_50_ WT PA14 = 1.25 and 1.30 respectively), whereas a third *gshB* insertion allele in the *ΔexoU* background (#6613) showed a considerably stronger attenuated phenotype (LT_50_ mutant/LT_50_ WT PA14 = 1.68) (Figure 5D). Construction of a series of double mutants carrying *ΔexoU* (or perhaps other mutations in the type III secretion apparatus or effectors) and mutations in other virulence loci would help to clarify the role of PA14 ExoU in pathogenesis of *C. elegans*.

#### Features of 41 PA14 tertiary set genes required for virulence in *C. elegans*

**Figure 6:**
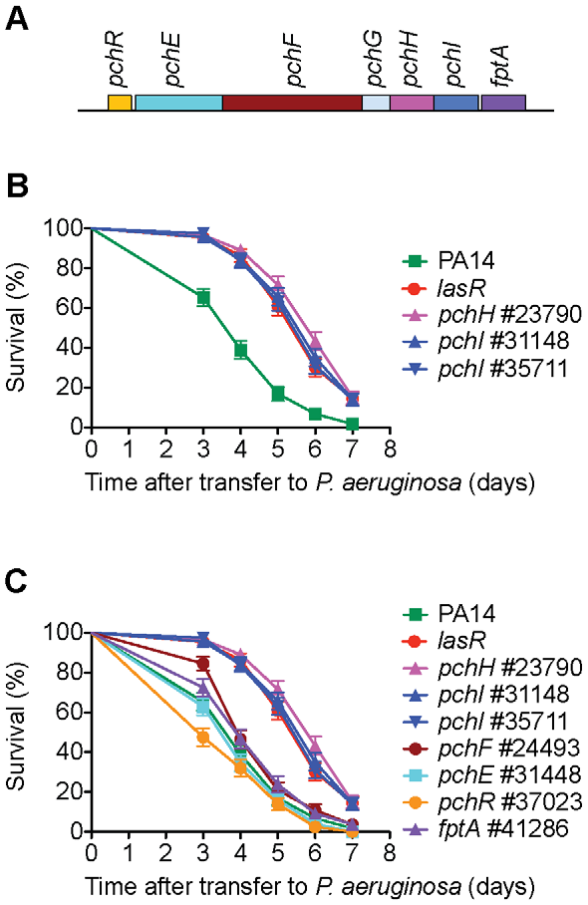
PchH and PchI but not pyochelin are required for normal virulence of PA14 in *C. elegans*. A) *pchH* and *pchI* encode putative ABC transporters with potential export functions and are the two terminal genes in a pyochelin biosynthetic operon [98]. B) Two *MAR2xT7* transposon insertions in *pchI* and the single available *MAR2xT7* allele of *pchH* are virulence-attenuated. C) Transposon insertion mutations in either the pyochelin biosynthetic genes *pchE* and *pchF* or the outer membrane transporter *fptA*, which transports pyochelin complexed with iron into the cell, have little effect on virulence. Mutations in *pchH* and *pchI* have been previously shown to produce wild-type levels of pyochelin in culture supernatant [98] and exhibit attenuated virulence in a neutropenic mouse model [99].

Figure 2 shows the relative virulence of the 41 mutants (corresponding to 41 genes) out of the 58 tested whose phenotypes were confirmed in the tertiary screen (Table S5, Table S6). Of the 41 genes, 21 have been previously implicated in *P. aeruginosa* virulence in at least one host and an additional 4 genes have been identified as virulence-related in other pathogens (Table 1). Thus 20 of the 41 (49%) genes identified in our screen are novel *P. aeruginosa* virulence-related genes. None of these 41 mutants exhibited significant growth defects and in 33 cases, the virulence-related phenotypes were verified by two or more independent *MAR2xT7* transposon insertions. In Figure 2, the ratio of the time to 50% nematode survival on each mutant to the 50% survival time on wild-type PA14 is presented (the average value of the ratio from multiple experiments is shown in most cases). The mutants for which killing assays were not repeated (g*acA, ptsP, vfR*) had been previously demonstrated to be virulence-attenuated in *C. elegans* in published studies [13], [54]. The ratios of 50% survival time of the positive *lasR* and *pilA* controls to WT PA14 were 1.63 (SEM = 0.08 for 10 independent experiments) and 1.22 (SEM = 0.04 for 10 independent experiments), respectively, and are shown as red bars in Figure 2. Among these 41 genes, five (indicated by “S” in Figure 2; *pchH*, *lysC*, PA1592, *aruG*, *fha2*) were represented by a single allele in the master library and the avirulent phenotypes corresponding to these insertions could not be verified by an independent insertion allele. The data shown in Figure 2 are for the mutant allele identified in the initial screen from the NR library (in the case where two alleles were identified a single allele was chosen). Examples of *C. elegans* survival curves (representative curves of assays repeated at least twice) from which the data in Figure 2 are derived are shown for *clpA* (Figure 4B), *gshB* (Figure 5D), *pchH* and *pchI* (Figure 6B), and *aruC*, *aruG*, *aruB*, *aruD* (Figure 3B). Additional survival curves are shown in the Supporting Information for *pepP* (Figure S5B), PA0456 (Figure S6B), *kinB* (Figure S7B), PA14_27700 (Figure S8B), PA0745 (Figure S9B), *vqsR* (Figure S10B) and *gshA* (Figure S11B).

**Table 1:** *P. aeruginosa* PA14 virulence-attenuated genes identified in the *C. elegans* infection model.

Locus	Description	*P. aeruginosa* host	Virulence-attenuated
*gacA*	two-component regulator	nematode, mouse[^13^]	
*Vfr*	cAMP dependent transcriptional regulator	mouse (PAK)[^109^]	
*pchH*	ABC transporter	slime mold, fly, mouse (22D10)[^99^]	
PA4005	conserved hypothetical protein		
PA14_27700	putative transcriptional regulator	nematode[^32^]	
*pepP*	proline aminopeptidase		
*hemK*	probable translation release factor methyltransferase		
*lysC*	aspartokinase		
*vqsR*	transcriptional regulator	nematode (TB toxin killing)[^108^]	
*kinB*	two-component sensor	zebrafish, mouse (PA01)[^86^], [^103^]	
*ptsP*	Phosphoenolpyruvate-protein phosphotransferase	nematode, mouse[^13^]	
*lasR*	HSL quorum sensing regulator	nematode, mouse[^13^], [^110^], [^111^]	
PA0745	putative enoyl-CoA hydratase isomerase	nematode (PA01 cyanide)[^54^]	
*rhlR*	HSL quorum sensing regulator	nematode[^111^]	
PA2550	putative acyl-CoA dehydrogenase		
*Mind*	cell division inhibitory membrane ATPase		*F. tularensis* [^112^]
PA1592	hypothetical protein		
*glnK*	nitrogen assimilation signal transduction protein		*S. typhimurium* [^76^]
*aruD*	arginine catabolism		
*gshA*	glutathione biosynthesis		
PA2015	putative isovaleryl-CoA dehydrogenase		
PA0456	cold shock domain protein		
*prpC*	methylcitrate cycle propionate metabolism	nematode (PA01 cyanide)[^54^]	*M. tuberculosis* [^113^]
*gacS*	two-component sensor kinase	nematode, mouse[^13]^, [^54^]	
*lasI*	quorum sensing HSL production	mouse (PA01)[^28^]	
*pchI*	ABC transporter	slime mold, fly, mouse[^99^]	
*aruG*	arginine catabolism		
*fabF1*	fatty acid biosynthesis	fly, mouse[^45^]	
*clpA*	ClpP protease chaperone & ATPase		*H. pylori* [^114^]
*aruB*	arginine catabolism		
PA1766	conserved hypothetical protein		
PA1216	hypothetical protein		
*pqsE*	quorum sensing regulation	mouse[^115^]	
*kdpD*	two-component sensor kinase		*S. typhimurium* [^76^]
PA1767	hypothetical cytoplasmic membrane protein		
*prpB*	methylcitrate cycle propionate metabolism	nematode (PA01 cyanide)[^54^]	
*fha2*	type VI secretion protein		
*gshB*	glutathione biosynthesis		
*aruC*	arginine catabolism		
*wbpL*	LPS O antigen synthesis	nematode, mouse[^32]^, [^33^]	
*pilA*	type IV pilus protein	nematode, mouse, human cells[^32]^, [116], [^117^]	
*pilF*	type IV pilus protein	nematode, mouse, human cells[^32]^, [116], [^117^]	
ORF_11	LPS O antigen synthesis	nematode, mouse[^32]^, [^33^]	

Genes are listed in descending order of contribution to virulence (according to the ratio of mutant LT_50_/wild-type LT_50_) using the data from Figure 2. Genes previously identified as required for normal levels of *P. aeruginosa* virulence in various model systems are indicated. In some cases only *P. aeruginosa* strains other than PA14 were examined and the strain and mode of killing is indicated in parentheses. The other pathogens, in which orthologs of these genes have been implicated in virulence, are noted in the last column.

#### Additional transposon mutants used to examine the role of identified operons

In bacteria, genes that function in common processes are frequently co-regulated in operons or clustered in the genome. We utilized the public PA14 database constructed in our laboratory [http://ausubellab.mgh.harvard.edu/cgi-bin/pa14] and BIOCYC [http://biocyc.org/PAER208963/NEW-IMAGE?object=Transcription-Units] to predict whether the genes corresponding to the avirulent mutants identified were in operons, based on annotation and the proximity and direction of transcription of adjacent genes. Of the 41 genes shown in Figure 2, our analysis suggested that 27 are located in 22 putative operons (see Figure 2). Multiple insertion mutations in three operons were identified in our screen, four in the *aru* operon (*aruC, aruG, aruD, aruB*), two in a pyochelin biosynthetic operon (*pchH, pchI*), and two (PA1766, PA1767) in an operon of unknown function. Utilizing the master insertion library of 24,000 mutants, we tested an additional 38 mutants (corresponding to 24 genes) in 11/22 of the putative operons and thereby identified six more genes in five operons that resulted in virulence attenuation when mutated: *aruF*, which is part of the *aru* operon; ORF_10, which had been isolated in a previous screen and together with ORF_11 forms a predicted transcription unit involved in O-antigen biosynthesis [32]; *pqsA*, which is required for synthesis of the quinolone quorum sensing molecule located upstream in the *pqsE* operon [55]; PA1218 and PA1221, which are upstream of PA1216 in an operon of unknown function; and PA4000 at the end of the operon containing PA4005. Note that mutations in *proA* and *nadD* directly upstream of PA4005 in the operon were identified in our primary screen but discarded because they are auxotrophs. Interestingly, in the case of 5/11 putative operons (containing *pepP, kinB*, PA0745, *clpA* and *fha2*) for which mutations in multiple genes were analyzed, only mutation of a single gene in the operon resulted in an attenuated phenotype. Two genes in the final operon examined (*pchH, pchI*) were identified in the primary and secondary screens, but mutations in the other genes in this pyochelin operon had no significant effect on virulence (Figure 6C). It is important to keep in mind that not all the genes in each of the operons examined were represented by *MAR2xT7* insertions in the master library.

Our finding that mutation of some but not all genes in an operon affect virulence may indicate redundant functions (perhaps located elsewhere in the genome) for those with no mutant phenotype or unique roles in virulence for specific genes within the operon. The *aru* operon, which encodes the catabolic enzymes for aerobic utilization of arginine as a carbon, nitrogen and energy source [56], [57], represented an unusual case where 5/5 genes in the *aruCFDGB* operon all exhibited a similar moderate attenuation of virulence when mutated. The *aru* insertion mutations did not observably affect growth of the bacteria on SK plates (although a single mutant in *aruF* #56181 had somewhat reduced growth on minimal media in the absence of nucleotides and amino acids) suggesting that the catabolism of arginine may be important for growth or virulence of the bacteria within the nematode. Interestingly, mutation of *aruE*, which is located downstream and adjacent to the *aruFGDB* operon, did not result in an avirulent phenotype. In the *P. aeruginosa* strain PA01 *aruE* is reported to be a separate transcription unit [57] (Figure 3).

### Virulence-Related Phenotypes of Selected Mutants

Seven putative virulence-related factors, cold shock domain protein PA0456, ABC transporters PchH and PchI, aminopeptidase PepP, putative enoyl-CoA hydratase/isomerase PA0745, ATPase/molecular chaperone ClpA, and putative transcriptional regulator PA14_27700 were chosen for further study. Mutants corresponding to these factors (*clpA*, Figure 4B; *pchH and pchI*, Figure 6B; *pepP*, Figure S5B; PA0456, Figure S6B; PA14_27700, Figure S8B; and PA0745, Figure S9B, C) all have a strong avirulent phenotype in *C. elegans*, exhibit normal growth kinetics *in vitro* (Figure S12), and represent genes whose role in *P. aeruginosa* virulence has not been previously characterized. The avirulent phenotype of all these mutants was confirmed with multiple transposon alleles except for *pchH* for which there is only a single allele available. In addition, in the case of PA0745, an in-frame deletion mutant was generated that was severely impaired in virulence, similar to the transposon allele #37629 isolated in the screen (Figure S9C).

Many of the genes previously identified as necessary for virulence of PA14 in *C. elegans*, for example those coding for the quorum sensing regulators RhlR and LasR, are known regulators of multiple virulence factors or virulence associated pathways [58], [59]. Both *lasR* and *rhlR* mutants have a spectrum of pigment and motility defects. *lasR* and *rhlR* mutants produce reduced levels of the blue-green pigment pyocyanin, *rhlR* produces no pyocyanin, and *lasR* mutants produce varying amounts dependent on conditions and growth phase [60]. Under certain growth conditions, *rhlR* and *lasR* mutants have been reported to produce less of the fluorescent siderophore pyoverdine [61]. *lasR* and *rhlR* mutants also exhibit dramatically reduced swarming motility [62], which is dependent on both the type IV pilus and the flagella and regulated by quorum sensing and a host of transcription factors [63].

We tested the seven selected virulence-related mutants for defects in motility (twitching, swimming and swarming assays) as well as for pyocyanin and pyoverdine production in comparison to *lasR* and *rhlR* mutants to determine whether they had a similar spectrum of defects and/or could be classified into groups based on common pigment or motility phenotypes (Table 2). It should be noted that of these phenotypes, only mutants in which type IV pilus function is affected have been shown to exhibit reduced virulence in the *C. elegans* infection model; pyocyanin does not appear to be necessary for virulence in the SK model and the roles of pyoverdine production, swimming and swarming have not been directly tested [12], [14]. Significantly, with the exception of PA14_27700, all of the mutants exhibited defects in some aspect of motility or pigment production. Mutation of putative cold shock protein PA0456 diminished pyocyanin production as did the quorum sensing regulators *lasR* and *rhlR*, whereas a *pepP* mutant had elevated pyocyanin levels. The putative enoyl-CoA hydratase/isomerase PA0745 produced reduced levels of pyoverdine. Among the tested mutants, 4/7 had clear swarming defects, but exhibited normal levels of swimming and twitching motility, suggesting that neither flagella nor type IV pili function was compromised. The *clpA* mutant was slightly attenuated for swarming and twitching, implying that there might be a type IV pilus defect in this mutant.

**Table 2:** Pigment and motility phenotypes of seven novel virulence mutants.

Strain	Pyocyanin	Pyoverdine	Twitching (cm)	Swimming (cm)	Swarming (SK)	Swarming (LB)
*lasR* (deletion)	0.06±0.01	1.10±0.18	0.49±0.02	2.30±0.12	±	−
*rhlR* (deletion)	0.00	1.11±0.17	0.53±0.01	2.53±0.09	−	−
PA0456 (#36116)	0.20±0.11	1.07±0.04	0.53±0.02	2.43±0.03	+	±
PA0745 (deletion)	1.05±0.04	0.33±0.04	0.56±0.02	2.32±0.05	±	±
*pchH* (#23790)	0.89±0.12	0.99±0.04	0.53±0.02	2.40±0.03	+	±
*pchI* (#35711)	1.12±0.05	1.09±0.07	0.60±0.01	2.30±0.10	+	±
*pepP* (#31907)	1.46±0.13	0.96±0.06	0.58±0.02	1.93±0.03	+++	+++
*clpA* (#39351)	0.92±0.01	0.88±0.05	0.48±0.02	2.30±0.12	+	+++
PA14_27700 (#32578)	0.98±0.03	0.92±0.06	0.58±0.02	2.40±0.00	+++	+++
PA14 WT	1.00	1.00	0.59±0.01	2.31±0.02	+++	+++

The average ratio of mutant to wild-type pyocyanin levels from four samples and the SEM is shown. The average ratio of mutant to wild-type pyoverdine levels from four samples and the SEM is shown. Twitching motility (1.5% LB agar) was measured as the radius of growth at the interface of the medium and the polystyrene plate and average radius and SEM from three inoculations is presented. Swimming motility was determined by the diameter of the turbid zone in semi-solid LB agar (0.35%) and average radius and SEM from three inoculations is presented. Swarming levels on the surface of 0.5% agar medium were qualitatively evaluated with number of and length of tendrils taken into account.

### Characterization of Genomic and Phylogenetic Distribution of Virulence-Attenuated Genes

The list of PA14 genes identified as being required for full virulence in *C. elegans* from the genome-wide screen provided the opportunity to examine the distribution within a species and conservation across bacterial species of a large set of genes required for virulence in a single host. We determined whether this set of virulence associated genes was biased towards *Pseudomonas* core or strain-specific (auxiliary) regions of the *P. aeruginosa* genome (as defined by Mathee et al. [41] and/or whether these virulence genes were preferentially located on genomic islands, as previously suggested for *Pseudomonas* virulence factors [39], [40]. In addition, we examined whether the PA14 virulence genes had a narrow phylogenetic distribution (unique to PA14, *P. aeruginosa*, *Pseudomonas*, or closely-related organisms) or were broadly distributed across prokaryotic phylogeny.

We used four sets of genes identified in our screen and a set of previously defined PA14 virulence genes downloaded from the Virulence Factor Database (VFDB) for all analyses. The sets of unique genes identified in the primary (294), secondary (170) and tertiary (41) virulence-attenuated screens outlined above and the auxotrophic genes identified in the primary screen but subsequently discarded (76) were used and for simplicity are referred to below as primary, secondary, tertiary, and auxotroph sets. All statistical analyses of the virulence genes identified in the *C. elegans* screen were done in comparison to the genes represented in the non-redundant (NR) library, as opposed to the entire PA14 genome, because this was the starting set for the screen. We used all four sets of genes in our comparisons to gain statistical power because the final set of 41 verified virulence-related genes was so small that most analyses did not make statistical cutoffs. In addition, using sets of genes from subsequent rounds of screening allowed us to look for enrichment that correlated with the refinement of the screen. To compare the virulence-related genes identified in our screen to previously identified *P. aeruginosa* genes, we made use of a set of 241 *P. aeruginosa* strain PA14 virulence genes downloaded from the Virulence Factor Database (VFDB) [52].

#### PA14 virulence-attenuated genes in the *P. aeruginosa* core and auxiliary (strain-specific) genome

**Figure 7:**
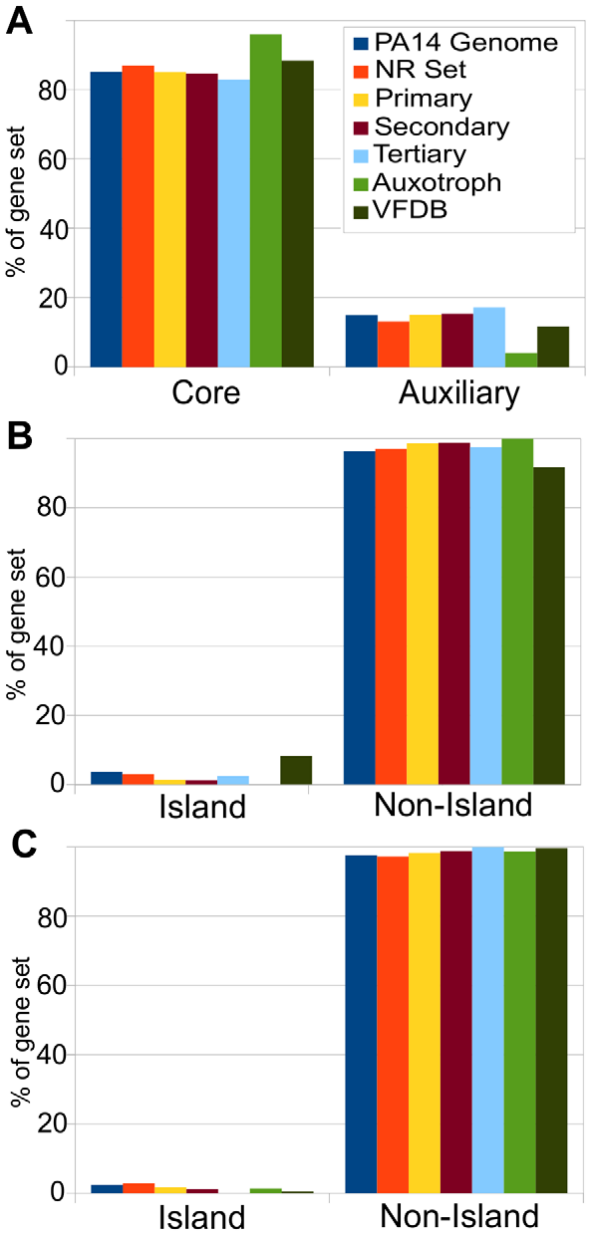
The distribution of *P. aeruginosa* PA14 genes required for virulence in *C. elegans* in the Core vs. Auxiliary genome and on both predicted and known genomic islands. A) The percentages of *P. aeruginosa* PA14 genomic genes, PA14-NR Set mutants, and primary, secondary, tertiary, auxotroph, and VFDB set genes that are part of the *P. aeruginosa* core and auxiliary genome as defined by Mathee et al. [41]. The auxotroph set is disproportionately part of the core genome. Genes in the primary, secondary, tertiary, and VFDB sets have proportions in the core and auxiliary genes that are statistically indistinguishable from the PA14 NR set and from the genome as a whole. B) The percentages of genes from the PA14 genome, the PA14-NR Set, and from the primary, secondary, tertiary, auxotroph, and VFDB sets that are located on genomic islands predicted by IslandViewer. Representation of primary, secondary, and tertiary gene sets on predicted islands was statistically representative of the genome as a whole and of the NR-set, whereas VFDB genes had a statistical overrepresentation of genes located on predicted genomic islands (p = 0.0005). C) The percentages of genes from the PA14 genomic, the PA14-NR Set, and from the primary, secondary, tertiary, auxotroph, and VFDB sets located on the known PAPI-1, PAPI-2, and PAGI-1 genomic islands. Representation of primary, secondary, and tertiary set genes was statistically identical to the genome as a whole and the NR-set. VFDB genes were statistically underrepresented on the known islands (p = 0.007). Refer to Table S7 for statistics.

Among the 5,893 annotated PA14 genes, 5,016 (85%) are core genes in that they are also present in *P. aeruginosa* strains PA01, PACS2, PA2192 and C3719, four *P. aeruginosa* clinical isolates [41]. The remaining 15% of the PA14 genes are designated as the auxiliary PA14 genome because they are genes that are missing in at least one of these five sequenced *P. aeruginosa* strains. The auxiliary genome is dispersed across the chromosome with regions of genomic plasticity containing strain-specific segments bordered by conserved genes. It should be noted that approximately 66% of the auxiliary genome is found in species outside of the *Pseudomonas* genus [41] and that the designated auxiliary and core genes may change somewhat over time as more *P. aeruginosa* genomes are sequenced. The distribution of the primary, secondary, and tertiary genes in the core versus auxiliary genome was statistically identical to the distribution of genes in the NR set. That is, roughly 15% of the virulence-related genes were part of the auxiliary genome (Figure 7A, Table S7). By comparison, we performed the same analysis with the auxotrophic genes and as expected for genes necessary for fundamental cellular metabolism, this set was significantly over-represented in the core genome (p-value = 0.009). Therefore, despite the fact that PA14 is much more virulent than strain PA01 in the *C. elegans* killing assay [32], our set of functionally defined PA14 virulence factors show no bias for the set of genes that is present in PA14 but absent in PAO1, consistent with our previous preliminary conclusion based on a much smaller data set [32]. Likewise, the representation of the VFDB genes in the core and auxiliary genomes was not significantly different from that of the NR set.

#### Location of PA14 virulence-attenuated genes on genomic island versus non-islands

We retrieved a list of predicted genomic islands of *P. aeruginosa* strain PA14 from the IslandViewer website [64] at (http://www.pathogenomics.sfu.ca/islandviewer/query.php). IslandViewer combines multiple methods of genomic island prediction. A list of PA14 genes located on the predicted islands was compiled. However, we noticed that the set of genes predicted to be on islands by IslandViewer only included 20 of the 143 genes located on the previously defined genomic islands PAPI-1, PAPI-2 and PAGI-1 [40], [65]. Therefore, an additional set of genes was compiled consisting of the 143 genes located on these known islands. We determined whether PA14 virulence genes identified in the *C. elegans* screen were associated with predicted genomic islands or with previously defined islands. Among the PA14 NR mutant set (3.0%; 131) of the genes represented are located on predicted genomic islands (similar to the 3.6%; 215 in entire PA14 genome). None of the identified auxotroph genes were located on predicted genomic islands and only one was located on a known genomic island. The virulence-related genes identified in our screen exhibited no bias for incorporation on the genomic islands predicted by IslandViewer nor on known genomic islands (PAPI-1, PAPI-2, and PAGI-1); in fact a non-statistically significant skew towards non-island regions of the genome was observed for the primary, secondary and tertiary sets in both comparisons. (Figure 7B,C, Table S7). This is in contrast to the significant overrepresentation of VFDB genes located in predicted genomic islands; *i.e.*, 8.3% of total VFDB genes compared to 3.6% of genes in the genome as a whole (Figure 7B). However, the VFDB virulence factors were also marginally underrepresented (1 gene, 0.4%, p-value = 0.017) on the known islands PAPI-1, PAPI-2 and PAGI-1 (Figure 7C, Table S7). The data above suggest that the virulence-related genes identified in the *C. elegans* SK model are not preferentially located in plastic regions of the *P. aeruginosa* genome.

#### Breadth of phylogenetic distribution of PA14 virulence genes across prokaryotes

Are the predominant contributors to *P. aeruginosa* PA14 virulence in *C. elegans* genes that are unique to PA14 or *P. aeruginosa* or are they common to many bacteria? The comparisons above suggest that they are not PA14 specific genes, but to ask the question more generally we used a variation of the phylostratigraphy approach [66] in which we evaluated the breadth of the phylogenetic distribution of each PA14 gene by examining 727 bacterial genomes for orthologs of these genes. Each gene was designated as belonging to one of seven breadth categories or phylostrata (0–6) depending on its distribution across the bacterial kingdom, with “0” representing genes that are only present in *P. aeruginosa* PA14, “1” for genes present in other *P. aeruginosa* strains but absent outside the species, “2” for genes occurring in other species in the genus *Pseudomonas* but absent outside the genus, and so forth, with the most broadly-distributed category “6” found in Archaea as well as bacteria (Figure S13; see Materials and Methods). It should be noted that each ORF is given a “breadth of phylogenetic distribution” designation based on the presence of a putative ortholog in the most distantly related organism and does not necessarily imply that orthologs for the given ORF are present in all groups less divergent than this most distantly related organism.

Whereas “breadth of phylogenetic distribution” may be a meaningful surrogate for the age of a gene in the case of eukaryotes, there is a significant pitfall to using it as such in prokaryotes, because horizontal gene transfer between unrelated prokaryotic lineages could distort the apparent age of a gene. An apparently young, narrowly distributed gene could be older than it seems if horizontally transferred from a previously unrelated unsequenced organism, but it would appear to be a new within the *P. aeruginosa* lineage. A more serious problem is presented by genes that appear older than they are, as a result of a limited number of horizontal gene transfer events between unrelated lineages. We reasoned that we could mitigate this latter complication by including information about the frequency of occurrence across the clades in which genes are represented. Broadly distributed genes that occur with high frequency across their phylogenetic breadths, have, we believe, a greater likelihood of being genuinely “old” genes. We therefore created a subset of our most broadly distributed genes, categories 4, 5, and 6, that are also represented in 50% or more of the sequenced genomes in the clades in which they occur. We dubbed this subset “high-frequency-broad-phylogeny” or HFBP genes, which comprise 6.9% of the NR Set. Since we were also interested in examining the representation of the newest or most recently acquired genes in *P. aeruginosa* strain PA14, we also considered genes that were *Pseudomonas*-genus-specific, which we dubbed “PGS” genes. For this set, we binned genes of breadths 0, 1, and 2, which total 9.6% of the NR set, reasoning that such a set would represent relatively new genes, while at the same time being a large enough set to provide some statistical power.

The distribution of phylogenetic breadths of all the PA14 genes, all the PA14 NR set mutants, and the primary, secondary, tertiary and auxotrophic set mutants is shown in Figure S13, with statistical analysis presented in Table S7. The virulence-attenuated primary, secondary, and tertiary sets of genes identified in the *C. elegans* screen show a trend of underrepresentation in the narrowest phylogenetic breadth classes, with the tertiary positives, the set that predominantly includes highly virulence-attenuated mutants, being most underrepresented in the narrow phylogenetic breadth classes. However, taken individually, these underrepresentations are not statistically significant, due to the small number of mutants in each set.

**Figure 8:**
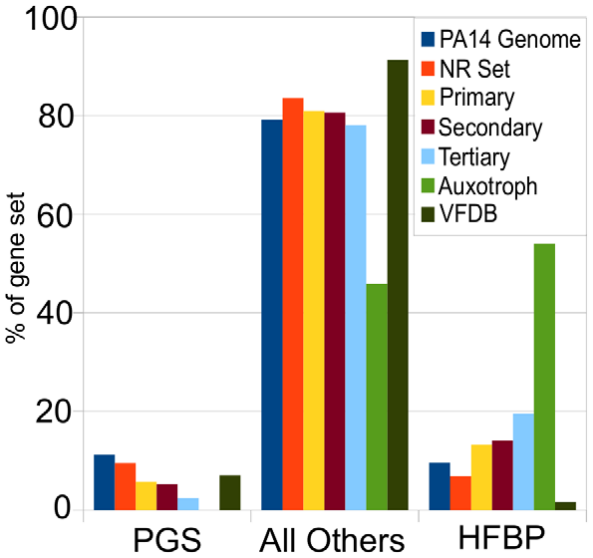
Among the PA14 genes required for virulence in *C. elegans*, “*Pseudomonas*-genus-specific” (PGS) genes are underrepresented, whereas “high-frequency-broad-phylogeny” (HFBP) genes are overrepresented. Based on phylostratigraphic analysis, PA14 genes required for virulence in *C. elegans* were classified as either “*Pseudomonas*-genus-specific” (PGS), presumably representing the newest genes in PA14, “high-frequency-broad-phylogeny” (HFBP), representing the oldest, most conserved genes in PA14, or “all others”. The percentage of each gene set, including the PA14 genome genes, the PA14-NR, primary, secondary, tertiary, auxotroph, and VFDB gene sets that are classified as PGS genes, HFBP genes, or all others genes, are shown. HFBP genes comprise 10% of the PA14 genome, and about 7% of the NR set genes. Furthermore, HFBP genes are increasingly overrepresented with successive iterations of the screen accounting for 13% of the primary set (p-value = 0.00004), 14% of the secondary set (p-value = 0.0005) and 19% of the tertiary set (p-value = 0.006). HFBP genes make up greater than 50% of the auxotroph set with a (p-value = 5.47×10^−28^) relative to the NR set. The PA14 VFDB set contains an underrepresentation of HFBP genes (1.6%, p-value = 0.0001). PGS genes make up 11% and 9.6% of the PA14 genome and NR set respectively. Over successive iterations of the screen, PGS genes become numerically more underrepresented relative to the NR set, comprising 5.7% of the primary set (5.7%, p-value = 0.01), 5.2% of the secondary set (p-value = 0.03, not statistically significant), and 2.4% of the tertiary set (p-value = 0.08, not statistically significant). Due to the small numbers of genes in the secondary and tertiary sets, only the underrepresentation in the primary set is significant after application of multiple comparison correction (FDR, q< = 0.05). PGS genes are underrepresented in the auxotroph set (0%, p-value = 0.0006). Statistical data for this figure are presented in supplemental Table S7.

When the same analysis was performed examining the HFBP and PGS gene sets in comparison with all the other PA14 genes (Figure 8, Table S7), we observed that there was a significant overrepresentation of HFBP genes among auxotroph set (p-value = 5.47×10^−28^) and in the primary, secondary and tertiary mutant sets (p-values of 0.00004, 0.0005, 0.006, respectively). At the same time, there was an underrepresentation of *Pseudomonas*-genus-specific (PGS) genes among the primary set (5.8% vs 9.6% in the NR Set, with p-value = 0.01), and the proportion of PGS genes appeared even more depleted among secondary and tertiary gene sets (5.3% and 2.4%), although the smaller size of these sets rendered these underrepresentations not statistically significant. However, we believe the statistically significant underrepresentation of PGS genes in the primary set, combined with the successive, increasing depletion of that category in the secondary and tertiary positives, suggests that those depletions are not spurious. In contrast to the gene sets from our screen, HFBP genes were underrepresented in the VFDB *P. aeruginosa* strain PA14 gene set (p-value = 0.00013). The apparent underrepresentation of *Pseudomonas*-genus-specific genes, most likely the youngest genes, and the overrepresentation of high-frequency-broad-phylogeny genes that likely represent older genes, point to a skew in the primary, secondary, and tertiary mutant sets toward older genes, and away from young, newly-acquired, or fast-evolving genes.

Taken together, these analyses of the genomic and phylogenetic distribution of virulence-attenuated genes demonstrate that the genes required for PA14 virulence in *C. elegans* are distributed throughout the PA14 genome on both predicted genomic island and non-island regions, are not unique to a particular *P. aeruginosa* strain and in fact are disproportionately potentially old genes with identifiable orthologs across a wide breadth of prokaryotic species.

## Discussion

### *P. aeruginosa* PA14 Genes Required for *C. elegans* Killing

We set out to define the spectrum of genes required for *P. aeruginosa* PA14 infection in a single host organism with the ultimate goal of elucidating the mechanisms underlying pathogenesis in this multi-host opportunistic pathogen. A genome-wide unbiased screen for *P. aeruginosa* strain PA14 mutants defective in killing *C. elegans* identified a set of 180 putative virulence-related mutants (corresponding to 170 genes) after two rounds of screening. The screen was validated by the isolation of mutants previously shown to be required for *P. aeruginosa* virulence in both nematodes and mammals or known to regulate processes or pathways linked to pathogenesis including, but not limited to, genes involved in quorum sensing, two component regulators of virulence, transcriptional regulators, genes involved in type IV pilus production, and O-antigen biosynthesis. Twenty genes in the 170 gene set overlapped with a set of previously defined virulence factors in VFDB, a database of *Pseudomonas*-related virulence factors. Overall, the PA14 genes identified in the genome-wide screen have an overrepresentation of highly conserved genes present in many bacterial phyla and are part of the stable *P. aeruginosa* genome, rather than being located on pathogenicity islands.

The set of 170 virulence-related genes is broadly distributed across 27 defined functional classes and DAVID GO term analysis and mapping onto KEGG pathways did not reveal any interpretable enrichment for particular functions or pathways. This breadth of functional classes parallels the virulence-attenuated mutants identified in an independent unbiased screen using signature tagged mutagenesis carried out by Potvin and coworkers in a rat chronic infection model [46]. Unlike the genes identified in our screen and by Potvin et al., virulence-related factors in the VFDB are enriched for secretion- and adherence-related proteins. A major difference between the virulence-related genes in VFDB and the genes identified in our unbiased screen is that many of the genes in VFDB were included because they encode secreted toxins, secretion systems, or cell surface structures [52], [67]. However, the sensitivity of our screen favored identification of mutants with strongly attenuated virulence. This was expected given the nature of the primary screen that required that both the parent nematodes live long enough to produce a significant brood and that the nematode brood mature on the mutant bacterial lawn. It is possible, therefore that mutants with a weak virulence-attenuated phenotype were not detected and this could potentially skew the collective analysis of virulence factors.

One explanation for why so few mutants identified in our screen correspond to secretion pathways or to secreted effectors is that many of *P. aeruginosa* virulence effectors appear to function redundantly in the *C. elegans* killing assay. In support of this conclusion, disruption of the ExoU cytotoxic phospholipase had no statistically significant impact on virulence, but appeared to create a sensitized background that allowed the detection of other relatively weak virulence factors. Further, McEwan et al. have shown that whereas PA14 exotoxin A (*toxA*) mutants have no or little defect in virulence, overexpression of ToxA in *E. coli* activates the worm immune system and ultimately kills an immune-compromised animal, suggesting that ToxA may play an active, but to date undetected, role in PA14 pathogenesis in the nematode host [68]. Similarly, Dunbar et al. [69] have shown that ToxA inhibits protein synthesis in *C. elegans* intestinal cells during an infection. ExoU and ToxA are secreted by distinct systems and the weak virulence attenuation of secretion system mutants in *C. elegans* implies that multiple secretion systems and effectors may contribute to virulence in *C. elegans* with no single system being paramount.

An alternative explanation for the identification of a limited number of secretion-related mutants in our screen may be linked to the fitness costs of maintaining a large set of effectors targeting a wide range of potential hosts. Only four Type III effectors have been identified in *P. aeruginosa*, whereas 46 families of effector proteins have been identified in various strains of the related plant pathogen *P. syringae* [70] and a typical *P. syringae* strain has 20 to 30 effectors [71]. In *P. syringae*, which has a much more limited host range than *P. aeruginosa*, type III effectors mostly target host defense signaling pathways and both enhance virulence in particular host plants, while eliciting a strong immune response in others [72]. This fact, combined with the observations that the genes encoding secreted effectors are often under diversifying selection [38], [73] and are typically located in plastic regions of the genome [70], [74], suggests that *P. syringae* strains actively co-evolve with a limited number of hosts. In contrast, from first principles, it seems highly unlikely that a broad host-range pathogen like *P. aeruginosa* PA14 can be simultaneously co-evolving with all of its multiple hosts. Therefore *P. aeruginosa* might employ a broader set of strategies to ensure survival in diverse hosts instead of maintaining large sets of host-specific virulence-related effectors.

In this context, a number of the strongly virulence-attenuated PA14 mutants identified in our screen may correspond to factors that enable survival of *P. aeruginosa* in the hostile environment of the *C. elegans* intestinal tract, which is acidic and filled with enzymes such as proteases, lipases, and DNAse that potentially disrupt bacteria [75]. Moreover, in response to pathogens, the nematode specifically upregulates transcription of many putative antimicrobial genes [18]. In order for *P. aeruginosa* to proliferate in the intestine and cause disease, it first has to survive. Both the two component potassium sensor KpdD and the nitrogen assimilation regulatory protein GlnK, identified in our screen, have recently been shown to play a role in the persistence of *S. typhimurium* in the *C. elegans* intestine and defects in outer membrane integrity may reduce the survival of *kdpD* and *glnK* mutants in the host [76]. In addition, the identification of two PA14 genes required for biosynthesis of glutathione, *gshA* and *gshB*, may be related to the role of glutathione as a protectant against stresses encountered in the worm intestine including reactive oxygen species and low pH [77]. Cold shock domain proteins, like PA0465 identified in our screen are another class of molecule that are induced by environmental stress and are generally thought to play a protective role in the cell [78].

Identification of a number of PA14 mutants corresponding to metabolic genes illustrates the importance of nutrient acquisition in virulence. Without specific biosynthetic or metabolic capabilities a pathogen may be unable to colonize or grow within a host. For example, *P. aeruginosa* mutants defective in purine biosynthesis are unable to replicate in neutropenic mice, presumably because the *in vivo* environment is deficient in purine [48]. In the cases of intercellular pathogens such as *Listeria monocytogenes* and *Mycobacterium tuberculosis*, specific amino acid and nucleotide auxotrophs are reduced in growth *in vivo* [79], [80]. We identified a number of metabolic genes including *prpB* and *prpC* and several *aru* genes that may be important for bacterial metabolism and growth under the nutrient conditions within the nematode intestine. Further, some of the putative virulence-attenuated mutants identified in the primary screen that were set aside for further study because they were determined to be auxotrophs (a typical step in many screens) might specifically reflect nutrient availability in the nematode intestine. In this regard it is notable that mutations in nine purine, five pyrimidine, and six tryptophan biosynthetic genes were identified in the primary screen for virulence-attenuated mutants, and although some of these mutants exhibited reduced growth on the killing assay medium, many did not have any observable difference from wild-type PA14, suggesting that the reduction in virulence might be due to aberrant growth of these auxotrophs *in vivo*.

The predominant contributors to PA14 virulence in our *C. elegans* infection based assay appear not to be individual effectors, but genes that regulate numerous effectors (like the quorum sensing regulators *lasR* and *rhlR*), genes that are vital for protecting the bacteria from the host defense onslaught, and genes that help *P. aeruginosa* obtain the necessary nutrients to survive in the host. Therefore the strategy of a broad host range opportunistic pathogen might fundamentally differ from a pathogen that targets specific hosts, relying more on multiple partially redundant secretion systems and their cognate effectors and strategies for survival under a wide-variety of metabolic and environmental conditions.

### Location of *P. aeruginosa* Virulence Factors in the Core *P. aeruginosa* Genome

The long-recognized association between virulence genes and regions of genomic plasticity, particularly genomic islands acquired by lateral transfer of genetic material [81], has been attributed to the competitive advantage conferred by horizontally-acquired virulence factors in an ongoing co-evolutionary struggle between a host and pathogen. Two pathogenicity islands carrying plant and animal virulence-related genes have been identified and characterized in *P. aeruginosa* PA14 (PAPI-1 and PAPI-2). Of the 11 genes located on PAPI-1 previously shown to be required for normal levels of virulence in plants and mice [40], only one (*rcsC*) was identified in our secondary screen as a weak mutant and it did not re-test in the tertiary screen. Overall our results showed no enrichment of virulence-associated genes on predicted genomic islands, or on the known genomic islands PAPI-1, PAPI-2, or PAGI-1. More generally, whether or not virulence genes in aggregate are preponderantly associated with genomic islands in *P. aeruginosa* has not been experimentally demonstrated. The statistical power of analysis with respect to the genomic locations of genes is limited in part by the fact that not all genes are expected to segregate independently. In this regard, it is worth noting that the apparent enrichment of *P. aeruginosa* VFDB genes on predicted genomic islands is primarily due to the cluster of functionally interdependent Type III secretion apparatus genes located between gene loci PA14_42440 and PA14_42660. In summary, our data and analysis of existing data suggest that neither *P. aeruginosa* VFDB genes nor the virulence-attenuated genes identified in our screen are preferentially found on genomic islands.

Roughly paralleling our observations with genomic islands, our analysis of the frequency of PA14 virulence genes in the core and auxiliary genomes (both from our screen and the VFDB) showed no statistically significant over or underrepresentation. The main difference between the core versus auxiliary genome distinction, and that of genomic islands, is that auxiliary genes include both genes that are lost from the genomes of some isolates, as well as those genes that are newly acquired. Genomic islands by contrast, specifically include only newly acquired genes. Taken together, these results suggest that there is no specific enrichment of *P. aeruginosa* virulence-related genes on islands or in strain-specific regions of the *P. aeruginosa* genome, at least with respect to those that are involved in the *C. elegans* slow killing assay.

To further test the hypothesis that the arsenal of *P. aeruginosa* virulence factors includes newly evolved and or newly acquired genes we investigated the phylogenetic breadth of distribution of the PA14 virulence genes, as well as the degree to which they belong to a set of putative old conserved genes, the so-called high-frequency-broad-phylogeny (HFBP) set, and probable newer genes, the *Pseudomonas*-genus-specific (PGS) set. We found that consistent with the result that virulence genes are not located on islands, that PA14 virulence genes involved in *C. elegans* killing are enriched in HFBP genes, which as a group are likely to be the most ancient and conserved prokaryotic genes. The vast majority of PA14 virulence genes appear not to be specific to the *Pseudomonas* genus, and in fact, such recently acquired or novel genes are underrepresented among our putative virulence genes. These observations underscore the point that many virulence factors with the most significant contribution to virulence can be old, highly conserved genes.

### Assessing the Generality of the *P. aeruginosa C. elegans* Infection Model

Although the *C. elegans* model, in which nematodes are “force-fed” a monoculture of *P. aeruginosa* [13], [43], [44], is somewhat artificial, so are other laboratory models of *P. aeruginosa* infection that require pricking of the body of a fly [45], lung inoculation with agarose beads in rats [46] and mice [47], and non-lethal cutaneous burns in mice [82]. The artificiality of these models and the need for a compromised host is in part dictated by the opportunistic nature of the pathogen. The fact that conserved immune defense pathways are activated in the *C. elegans* host by *P. aeruginosa* strongly supports the view that the nematode is responding to *P. aeruginosa* as a pathogen [83]. Although we don't yet know whether *P. aeruginosa* is a natural pathogen of *C. elegans*, since both organisms live in the soil, it is likely that they encounter one another, and it is not inconceivable that the pathogenicity interactions that we observe may approximate the interactions of *P. aeruginosa* with weakened individual *C. elegans* animals in the wild.

Although some putative *P. aeruginosa* virulence factors are common to both mammalian and *C. elegans* host models, the degree to which *P. aeruginosa* virulence factors are shared, and implicitly, the degree to which *P. aeruginosa* virulence strategies are common to *C. elegans* and mammalian hosts is not yet clear. That only a single putative virulence-related gene is shared between the 170 virulence-attenuated genes from our secondary set, the rat chronic infection set defined by Potvin et al., and the VFDB set, argues against the idea of a core set of virulence factors common to all infection models. The spectrum of virulence factors that play a role in a given host model is likely to depend on a wide variety of factors including the characteristics of the site of infection, such as pH, ionic strength, nutrient availability, and temperature, the type of immune compromise, the phase of infection, and the particulars of the immune response, including the presence of host factors, and even host behavior. Indeed, two additional PA14 pathogenicity assays in *C. elegans* have been developed in our laboratory, a toxin-mediated killing model [12], [14] and a liquid killing assay (N. Kirienko and F. Ausubel, unpublished). PA14 appears to utilize distinct mostly non-overlapping sets of virulence-related genes to kill nematodes in the three different models. Nevertheless, all three of these assays have identified virulence factors that play important roles in various aspects of mammalian pathogenesis. The implication for human pathology is that the predominant virulence factors that play a role in different types of *P. aeruginosa* infection in humans may be somewhat distinct.

### Implications for Multi-host Opportunistic Pathogens

In summary, the data from our unbiased genome-wide screen for *P. aeruginosa* virulence factors involved in *C. elegans* killing in a specific infection model, suggest that in comparison to host-specific pathogens, *P. aeruginosa* may employ a smaller arsenal of host-specific effectors, and rely more on conserved, generic virulence factors and on its ability to endure host defense responses. While it is not yet clear that this same strategy is employed by multi-host pathogens beyond *P. aeruginosa* that are capable of infecting organisms from multiple phylogenetic kingdoms, this may explain why the major genes contributing to PA14 virulence in *C. elegans* are not overrepresented on genomic islands, are not PA14 or *P. aeruginosa* specific genes, and may in fact be biased for ancient genes common to many other prokaryotic species. These observations are consistent with the view of *P. aeruginosa* PA14 as a generalist pathogen for which the relationship with *C. elegans* is opportunistic rather than co-evolved. Indeed it is likely that no significant co-evolution occurs between *P. aeruginosa* and any of the hosts for which it is an opportunistic pathogen, both because of the rarity of pathogenic interaction, and because of the likelihood that co-evolution with multiple hosts would necessitate balancing opposing evolutionary pressures from those hosts. From a clinical perspective, the multiplicity and apparent combinatorial nature of *P. aeruginosa's* virulence factors may pose a challenge for the development of new therapeutics to fight *Pseudomonas* infection. This work does not settle the question of whether the profile of virulence factors of a multi-host pathogen is likely to be different from that of host-specific pathogens in terms of the reliance on conserved effectors that target highly conserved features of eukaryotic biology, but it is a question that deserves further inquiry.

## Materials and Methods

### Bacterial Strains

*P. aeruginosa* strain PA14 mutants are gentamycin resistant *MAR2xT7* transposon insertion mutants unless otherwise stated [42]. The *lasR* mutant used as a control was a deletion that removes the *lasR* ATG and carries a gentamycin cassette [84]. Similarly, the *rhlR* mutant used as a control in the pigment and motility assays was a deletion mutant that carries a gentamycin cassette [84]. These mutants have an identical avirulent phenotype to subsequently generated clean in-frame PA14 *ΔlasR* and *ΔrhlR* mutants (data not shown). The *pilA* mutant is a tetracycline resistant Tn5-B30 transposon insertion mutant provided by G. O'Toole [85]. ΔPA0745 is a complete in-frame deletion of the ORF with a concomitant insertion of a *Pac*I restriction site generated by the method previously described in Chand et. al. [86]. Unless otherwise indicated, *MAR2xT7* mutant bacteria were streaked from frozen stocks onto LB agar containing 15 µg/ml gentamycin to isolate single colonies used for inoculation. WT PA14 (as well as *lasR* and *pilA* mutants) was streaked on LB agar containing 100 µg/ml rifampicin.

### Nematode Strains

N2 Bristol L4 animals were used for the primary and secondary screens. Tertiary tests were done with CF512 *fer-15(b26)II;fem-1(hc17)IV* temperature sensitive sterile nematodes. CF512 worms were propagated at 15°C, egg-prepped and hatched overnight at 20°C in M9 liquid media in the absence of food. Starved L1 animals were dropped onto NGM plates seeded with standard *E. coli* OP50 and grown for approximately 20 hours at 15°C and then 20 more hours at 25°C to generate staged L4 sterile animals for experimentation.

### Primary Screen

We used the non-redundant PA14 *MAR2xT7* transposon library of ordered mutants to screen for avirulent mutants [42]. The entire library was screened twice with the exception of a single 96-well plate of mutants (plate 14.3) that was not screened because it contained mostly known slow-growing mutants. Bacteria were inoculated from the frozen 96-well stock plates into 150 µl of LB in 0.5 ml 96-well Masterblocks (Greiner) and grown overnight with shaking for 16 hours at 37°C. 10 µl of each culture was spotted onto slow killing (SK) agar (standard nematode growth media, NGM, containing 0.35% instead of 0.25% peptone) in each of two wells of a 6-well culture plate (Falcon) [12]. Two previously identified virulence-attenuated mutants, quorum sensing regulator *lasR* (highly attenuated) and typeIV pilin *pilA* (moderately attenuated) were included as positive controls in the screen. Plates were scored qualitatively after 4 days at 25°C and designated as either “strong”, indicating large numbers of gravid animals (generally on *lasR* mutant bacteria hundreds of gravid nematodes were present and the bacterial lawn was mostly or completely consumed) or “weak”, indicating an observable increase in the number of worms or gravid adults present compared to PA14 (*pilA* usually resulted in an increase in the total brood size with a bias towards older stage larvae and gravid adults; the *pilA* control wells exhibited variability in the number of progeny alive and were near the limit of sensitivity in the screen). Each mutant was screened blind on two separate days for a total of four wells screened per mutant. Mutants that were scored as strong in either one or both repetitions or weak in both were retained for a total of 399 primary mutants. Approximately half way through the primary screen it became clear that lawn growth phenotype was relevant to our study, and from then on we scored for lawn growth. We therefore have a crude assessment of growth for about 50% of the mutants identified in the primary screen.

### Auxotroph Screen

All 399 of the mutants from the primary screen were streaked from the frozen library stock onto LB agar plates containing 15 µg/ml gentamycin and grown overnight at 37°C. To quickly test for potential auxotrophs, a single colony from each mutant was picked and streaked on 1) Neidhardt supplemented MOPS defined media without amino acids or nucleotides (TEKNOVA EZ Rich Media), 2) Neidhardt supplemented MOPS defined media plus amino acids and nucleotides, and 3) LB containing 15 µg/ml gentamycin agar plates. Plates were incubated overnight at 37°C. 86 mutants that either exhibited no growth on minimal media minus supplements or significantly reduced growth were removed from the pool of putative avirulent mutants and not examined in further rounds of screening (Table S2). 15/56 of the auxotroph mutants for which a plate growth phenotype had been annotated (see above) had obvious growth defects on SK agar plates.

### Secondary Screen

The 313 mutants from the primary set that exhibited normal growth on minimal media without addition of amino acids and nucleotides (Table S3) were screened for attenuation of virulence in standard SK assays and scored for both killing of parental (P_0_) animals and the number and maturity of worm progeny produced [13]. A single bacterial colony was inoculated into 5 ml of LB media and grown for 14.5 hours at 37°C with aeration on a rotating wheel. 10 µl of this overnight culture was spread onto each of two SK agar plates (3.5 cm culture plates (Falcon), plates were incubated for 24 hours at 37°C, and then 24 hours at 25°C. 30–40 N2 L4 animals (raised on standard NGM plates with *E. coli* OP50 as food) were picked to each SK plate and the plates were incubated at 25°C for a total of 60–80 animals per assay. Live and dead animals were counted 2–3 times over 1–3 days after transfer to the pathogen. Animals were considered dead if they did not respond to a gentle touch and were removed from the plate. After 4 days at 25°C, the number and age of the nematode progeny on each plate was qualitatively assessed as compared to those on PA14 WT. Secondary positives were ranked as “strong” (strongly virulence-attenuated similar to the *lasR* mutant), “moderate” (less attenuated than *lasR* but greater than or equal to *pilA*) and “weak” (less attenuated than *pilA* for parental killing but clearly exhibiting an increase in progeny number and age over WT PA14). 180 *MAR2xT7* mutants exhibited either attenuation of P_0_ parental killing or allowed increased production and development of nematode progeny (Table S4). The identity of the *MAR2xT7* mutants was confirmed by sequencing of arbitrary PCR products as previously described [42], see Table S4.

### Tertiary Screen

58 *MAR2xT7* mutants were re-screened in standard SK assays using sterile *fer-15(b26)I:fem-1(hc17)IV* animals (Table S5). Bacteria were grown as indicated above for the secondary screen. Three 3.5 cm SK agar plates were seeded with each bacterial strain and 35–50 L4 worms were transferred to each plate for a total of 100–150 animals per assay. Live and dead animals were counted every day over approximately 7 days. PA14 WT and *lasR* were included in each assay as controls. The resulting 41 virulence-attenuated mutants shown in Figure 2 were all tested in at least two separate experiments unless otherwise indicated. Statistical analysis of the curves summarized in Figure 2 was done using Prism 5 Log-rank (Mantel-Cox) test and the difference between the mutant and wild-type curves was highly significant in all cases with a p-value<0.0001. Time to 50% survival was calculated (Prism 5.0 linear regression Hill equation LogEC50). All killing assays presented in the manuscript (Figures 3, 4, 5, 6, S5, S6, S7, S8, S9, S10, and S11) are a representative example of two or more experiments that resulted in the summary shown in Figure 2.

### Assessment of Growth

Growth of bacterial mutants was evaluated by four methods: 1) Overnight cultures inoculated from a single colony into 5 ml of LB and grown for 14.5 hours at 37°C with shaking were visually compared to wild-type PA14 grown under the same conditions. Mutants that were observably less turbid were considered slow growers (in the case of PA14_45650 mutant #54246 the cells appeared to be lysed). 2) Slow killing agar plates spread with each mutant were examined prior to transfer of worms. Bacterial lawns that were thin or exhibited other aberrant phenotypes (*nusA* mutant #55834 had large colonies that emerged on top of the lawn) were removed from the mutant pool. 3) Many of the secondary and tertiary positive mutants were grown overnight in LB and M63 minimal media in a 96-well plate without shaking and the OD_600_ was measured every 15 minutes for 15 hours at 37°C in a Molecular Devices Spectra Max M5. The rate of growth during two hours of maximal growth was compared to WT (Table S5). 4) The growth of nine mutants (*pchH*, *pchI*, PA14_27700, PA2550, PA0456, PA0745, *clpA*, PA1216, *vqsR*) in 5 ml of M63 minimal in a standard culture tube on a rotation wheel at 37°C was measured by counting colony forming units. All nine of these mutants grew as well as WT (Figure S12).

### Pyocyanin Assay

A pyocyanin assay was modified from Essar et. al. 1990 [87]. A single colony of WT or mutant bacteria was inoculated into 5 ml of LB media and grown for 16 hours at 37°C on a rotating wheel. 1 ml of saturated overnight culture was transferred to a microfuge tube and cells were pelleted by centrifugation at 14,000 RPM for 2 minutes. 800 µl of the supernatant was transferred to a new tube, extracted with 600 µl of chloroform and the phases were separated by centrifugation for 5 minutes at 14,000 RPM. The chloroform phase was then re-extracted with 0.3 ml of 0.2 N hydrochloric acid. The pyocyanin content of 100 µl of the aqueous acidic phase was quantitated based on absorbance at 520 nm. The A_520_ was normalized to cell number (A_600_ of the original overnight culture). A Δ*phzA1-G1*/Δ*phzA2-G2* [88] mutant that does not produce any phenazines had no detectable A_520_. The pyocyanin produced by each bacterial strain was measured from the growth of two individual colonies on two separate days. For each of the four cultures, the ratio of A_520_ (normalized to cell number as measured by A_600_) of mutant to WT was calculated and the average of these four ratios is shown in Table 2. The error presented is the SEM of the four mutant/WT ratios.

### Pyoverdine Assay

A single colony of WT or mutant bacteria was inoculated into 5 ml of M9 media and grown overnight for 18 hours at 37°C on a rotating wheel. Cells were pelleted by centrifugation at 14,000 RPM for 2 minutes in a microfuge tube and the supernatant was diluted 10 fold in 10 mM Tris pH 7.4. Pyoverdine content was determined by measurement of fluorescence (400 nm excitation, 460 nm emission) [89], [90]. No fluorescence above background was detected in pyoverdine biosynthetic mutants, *pvdD* (#40342) and *pvdA* (#30448). The pyoverdine produced by each bacterial strain was measured from the outgrowth of two individual colonies on two separate days. For each of the four cultures, the ratio of fluorescence (normalized to cell number as measured by A_600_) of mutant to WT was calculated and the average of these four ratios is shown in Table 2. The error presented is the SEM of the four mutant/WT ratios.

### Swarming Assay

The swarming assay was modified from Overhage (2008) [91]. A single colony of WT or mutant bacteria was inoculated into 5 ml of LB media and grown for 15 hours at 37°C with aeration. 2 µl of each overnight culture was spotted onto the surface of LB 0.5% agar and SK 0.5% agar plates and then incubated at 37°C overnight. Each mutant was tested in triplicate on two separate days. As expected, the *rhlR* mutant (deficient in rhamnolipid production) and *pilA* mutant (typeIV pilin) were defective in swarming [62]. Mutants were visually compared to WT PA14 and were qualitatively evaluated for swarming radius and number of tendrils (Table 2).

### Swimming Motility

The swimming motility assay was based on Darzins (1993) [92]. A single bacterial colony was picked with a straight end loop and inoculated into LB swim agar (0.35% agar). Plates were incubated 8–12 hours at 37°C. The diameter of the flagellum-mediated motility generated turbid zone was measured. Each mutant was tested in triplicate and the average with SEM is presented in Table 2.

### Twitching Motility Assay

The twitching motility assay was based on O'Toole 1998 [85]. A portion of a single bacterial colony was picked with a straight end inoculation loop and stabbed to the bottom of a LB agar plate (1.5% agar). Plates were incubated overnight at 37°C and then 2 days at room temperature. The growth at the interface between the agar and the polystyrene plate (radius from the inoculation point) was measured. The *pilA* mutant exhibited no twitching motility. Each mutant was tested in triplicate and the average with SEM is presented in Table 2.

### Breadth of Phylogenetic Distribution of *P. aeruginosa* strain PA14 Genes Using a Variation of the Method of Phylostratigraphy [66]

A database of orthologs of *P. aeruginosa* strain PA14 genes across 727 sequenced prokaryotic genomes (including PA14) was created. Finished microbial genome sequences were obtained as downloaded packages from the NCBI ftp site (ftp://ftp.ncbi.nlm.nih.gov/genomes/Bacteria/) on August 25, 2008. PA14 proteins were used as BLASTP queries against each bacterial genome. Putative orthologs were reciprocal best hits against the corresponding proteins in the subject genomes. Blast results against subject genomes were required to have an e-value equal to or less than 0.0001. Reciprocal blasts against the PA14 genome were required to have e-values of 0.001 or less. Putative orthologs were required to align for at least 80 percent of their length and have less than 20% difference in protein sequence lengths, thereby conserving overall domain structure. The e-value constraint was permissive to allow detection of distant orthologs, but the requirement for alignment length was fairly stringent. Breadth of phylogenetic distribution, also called phylostratum, was a measure of the maximal phylogenetic distance at which an ortholog occurs. Breadth was defined as: 0 for proteins specific to PA14, 1 for proteins that occur in multiple strains of *Pseudomonas aeruginosa*, but not in other species, 2 for proteins that occur in multiple *Pseudomonas* species but not in other genera, 3 if across gamma and beta proteobacteria, 4 if across proteobacteria, 5 if across eubacteria, and 6 if across eubacteria and archaea (Figure S13).

### Statistics

P-values for overrepresentation and underrepresentation were calculated as Fisher Exact Test right and left probabilities, respectively, using version 1.21 of the Text::NSP::Measures::2D::Fisher Perl module, available from CPAN.org [93]. Multiple comparison correction using False Discovery Rate (FDR) was performed where indicated [94]. The maximal value of q was 0.05.

### KEGG Pathway and GO Term Analysis

Genes of *P. aeruginosa* strain PA14 were mapped to KEGG pathways using the KEGG Mapper program (http://www.genome.jp/kegg/tool/map_pathway1.html). The total number of mutants in each pathway was summed for those genes in the PA14 NR set and for each mutant set analyzed. P-values for overrepresentation of each pathway were calculated using Fisher exact test right-probabilities, and their significance was assessed using the Bonferroni correction [94], [95]. GO term analysis was performed using DAVID (http://david.abcc.ncifcrf.gov/home.jsp) using the genes in the NR set as a background, and the genes in the sets of analyzed positives as gene-lists. The DAVID software package calculates p-values for each GO term automatically, and also gives an EASE score, which was used to assess the significance of the overrepresentation of any given GO term.

## Supporting Information

Figure S1 **Flow chart of screening procedure for virulence-attenuated PA14 transposon mutants in** ***C. elegans*** **.**
(TIF)

Figure S2 **Correlation of virulence attenuation in standard SK and progeny survival assays.** A) *lasR*, *gacA*, *ptsP*, *mucD*, *rhlR* and *pilA* mutants all have attenuated virulence in a standard slow killing (SK) assay (60–80 N2 worms tested). B) The number and developmental stage of the progeny on the SK plates in panel (A) four days after transfer of the parent worms to pathogen at 25°C. On PA14, there were few progeny and no gravid adults were observed whereas gravid adult progeny were found on the virulence-attenuated *P. aeruginosa* strains. The number and age of progeny on the mutant plates was qualitatively scored in comparison to the PA14 WT plates. Plates seeded with *gacA* and *ptsP* were overrun with hundreds of gravid adult worms that consumed the bacterial lawn. *lasR*, *mucD*, *rhlR* and *pilA* plates contained fewer gravid adult worms but all were observably greater than plates seeded with WT PA14.
(TIF)

Figure S3 **Percentage of mutants that are VFDB genes.** The percentage of primary, secondary, and tertiary, and auxotroph set virulence-attenuated genes that are VFDB and non-VFDB genes are indicated. VFDB genes are significantly overrepresented in the primary, secondary, and tertiary sets, with p-values of 5.5×10^−6^, 2.1×10^−5^, and 3.7×10^−5^, respectively. Furthermore, their overrepresentation increases with successive screen iterations.
(TIF)

Figure S4 **Venn diagram showing the overlaps between the 170 virulence-attenuated genes obtained in the secondary screen, the Potvin set, and the PA14 VFDB set.**
(TIF)

Figure S5 **Multiple** ***pepP*** **alleles but not alleles of other genes in the operon have attenuated virulence.** A) PepP is a cytoplasmic aminopeptidase that cleaves aminoacyl proline dipeptides from the N terminus of polypeptides. In PA14 *pepP* appears to be the second gene in a 5 gene operon comprised of PA5225, a hypothetical protein, *pepP*, *ubiH* a ubiquinone biosynthetic enzyme, PA5225 another hypothetical protein, and terminating with PA5221, an ORF with homology to *E. coli visC* a pyridine nucleotide-disulphide oxidoreductase in the *ubiH* family. B) Four *MAR2xT7* alleles of *pepP* display highly attenuated *C. elegans* killing. C) Transposon mutants in 4/5 genes in the *pepP* operon were tested for their effect on virulence (no mutant was available in PA5221). Mutants in the two downstream genes tested, *ubiH* and PA5222 exhibited wild-type levels of virulence whereas the single mutant in the upstream PA5225 had a modest attenuation of virulence (compared to *pepP*) that might be due to effects on *pepP* expression. *pepP* mutant #31097 produced elevated levels of pyocyanin and had somewhat reduced swimming ability, although swarming was normal on both SK and LB (Table 2). The PepP proline aminopeptidase could function in utilization of exogenous peptides as nutrients, degradation of proteins or protein maturation, perhaps of a specific substrate relevant to virulence. There is a precedent for aminopeptidase function being linked to virulence associated phenotypes. *P. aeruginosa* PepA was shown to be involved in the regulation of alginate biosynthesis [100].
(TIF)

Figure S6 **Mutations in cold shock domain (CSD) protein PA0456 are virulence-attenuated in** ***C. elegans*** **but mutations in four other CSD proteins do not affect virulence.** A) PA0456 is most likely a single gene transcription unit. PA0456 (previously annotated as *cspB* in the PA14 genome) contains a canonical cold shock domain (CSD) and is homologous to other CSD proteins (57% identical to the major *E. coli* cold shock protein, CspA, and 65% to *B. subtilis* CspB as determined by BLASTP). Although the first cold shock proteins (Csps), a conserved family of small mostly acidic proteins that bind single stranded DNA and RNA, were identified as major proteins induced upon temperature downshift, some members of the family are not induced upon cold shock and many are implicated in other cellular functions [101]. B) Three independent *MAR2xT7* transposon insertions in PA0456 have attenuated virulence in *C. elegans*. C) Transposon insertion mutants in four additional CSD containing genes (*capB* PA3266, PA0961, *cspD* PA2622 and PA1960) have wild-type virulence in *C. elegans*. Five additional CSD-containing homologs of PA0456 (PA3266, PA1159, PA0961, PA2622, PA1960) were identified by BLASTP against the PA14 protein database with 76, 66, 62, 54 and 41% identity to PA0456 respectively; all 5 contain a CSD as analyzed by Prosite and transposon insertion mutants were available in 4/5 of these genes. Among the CSD containing proteins tested, PA0456 appears to be unique in its role in virulence, suggesting that the cold shock response per se is most likely not required for virulence of PA14. PA14 PA0456 has been reported to be regulated by quorum sensing [102], and in keeping with these findings PA0456 exhibited defects in quorum sensing regulated phenotypes; the PA0456 mutant had reduced pyocyanin production and swarming motility (Table 2).
(TIF)

Figure S7 **KinB (PA5484) sensor kinase is required for PA14 virulence in** ***C. elegans*** **.** KinB negatively regulates alginate production and has been recently shown to be required for virulence in zebrafish embryos and mice [86], [103]. A) *kinB* and its cognate response regulator *algB* form a two gene operon. B) Two *MAR2xT7* transposon alleles in *kinB* (as well as an in-frame deletion of *kinB* described in Chand et al., data not shown) are reduced in virulence. C) Transposon mutants in *algB* (and an in-frame deletion of *algB* described in Chand el al., data not shown) exhibited wild-type virulence in *C. elegans*, paralleling their lack of phenotype in zebrafish [86].
(TIF)

Figure S8 **Mutations in putative transcriptional regulator PA14_27700 and possible ECF sigma factor PA14_27690 are virulence-attenuated in** ***C. elegans*** **.** A) PA14_27700, a Crp/FNR-type transcriptional regulator that appears to have no homologue in PA01 [32], is located 264 bp downstream of PA2817 and 66 upstream of PA14_26790, a FecI-like extracytoplasmic function (ECF) sigma that also has no PA01 homologue. It is unclear whether PA14_27700 and PA14_26790 form an operon. B) Multiple *MAR2xT7* insertions in both PA14_27700 and PA14_27690 are attenuated in virulence. Although not initially identified in our screen, mutants in PA14_26790 have a strong virulence-attenuated phenotype like PA14_27700. ECF sigma factors, to which PA14_26790 has homology, are commonly co-transcribed with a regulatory anti-sigma factor and the ECF sigma factor and its regulatory anti-sigma factor frequently play an important role in adaptation to the external environment [104]. Canonical transmembrane anti-sigma factors have a small cytoplasmic regulatory/inhibitory domain linked by a transmembrane domain to a C-terminus sensory domain that resides in the periplasm. PA14_27700 has neither a signal sequence nor a transmembrane domain suggesting that it functions in the cytoplasm. However, most anti-sigma factors are poorly conserved at the sequence level and there are anti-sigma factors that sense cytoplasmic stimuli. PA14_27700 #32578, although attenuated in virulence, does not exhibit any obvious defects in pigment production or motility in our assays (Table 2).
(TIF)

Figure S9 **The virulence-attenuated phenotype of** ***MAR2xT7*** **insertions in putative enoyl-CoA hydratase isomerase PA0745 is recapitulated by an in-frame deletion and is not dependent on pyoverdine production.** A) PA0745, a putative enoyl-CoA hydratase isomerase, is the third gene of what is most likely a 5 gene operon: PA0747 a probable aldehyde dehydrogenase, PA0746 a putative acyl-CoA dehydrogenase, PA0745, PA0744 another putative enoyl-CoA hydratase/isomerase, and PA0743 a probable 3-hydroxyisobutyrate dehydrogenase. B) Insertions in PA0745 exhibit a virulence-attenuated phenotype but mutants of PA0746, the only other gene in the putative operon for which *MAR2xT7* mutants were available, exhibited wild-type levels of virulence. C) The virulence-attenuated phenotype of an in-frame deletion mutant of PA0745 is complemented by expression of the entire operon (PA0747-PA0743) in trans. The operon was expressed under its own promoter; PA14 DNA from genome position 4851671–4848075 was cloned in Pseudomonas vector pucP19 [105]. D) A defect in pyoverdine production in PA0745 mutants (Table 2) is not the cause of the PA0745 avirulent phenotype. Pyoverdine biosynthetic mutants *pvdA* and *pvdD* show minimal to no attenuation of virulence. It has been suggested that PA0745 enoyl-CoA hydratase isomerase may function in the production of cis-2-decenoic acid, a fatty acid signal responsible for inducing biofilm dispersal, due to its weak homology to RpfF which produces cis-11-methyl-2-dodecenoic acid, the diffusible soluble factor (DSF) required for virulence in *Xanthomonas campestris* [106], [107]. However, PA0745 and *X. campestris paaF* (required for the breakdown of phenlyacetic acid) are reciprocal top BLASTP hits with 65% identity over 95% of the protein. The putative enzymatic functions of the genes in the PA0745 operon suggests that this operon may collectively synthesize or degrade an as yet unidentified product/products that affect siderophore production, control of swarming, and virulence in *P. aeruginosa*.
(TIF)

Figure S10 **Transposon mutants in transcriptional regulator** ***vqsR*** **, but not genes in the adjacent downstream operon, are attenuated in virulence.** VqsR (PA2591) is a LuxR type transcriptional regulator that controls expression of quorum sensing and virulence genes. A *vqsR* mutant of *P. aeruginosa* strain TB has been shown to be impaired in homoserine lactone production and is attenuated in a liquid *C. elegans* killing assay that occurs over a period of hours, similar in kinetics to toxin mediated PA14 “fast-killing” [108]. A) *P. aeruginosa* PA14 *vqsR* appears to be transcribed as an individual ORF; it is located 120 bp upstream of an operon that encodes two conserved hypothetical proteins, PA2590 a putative outer membrane receptor protein and PA2589 a possible permease. B) Two *MAR2xT7 vqsR* transposon mutants have reduced virulence in *C. elegans*. C) *MAR2xT7* mutants in the operon adjacent to and downstream of *vqsR* (PA2590 and PA2589) are as virulent as WT PA14. In keeping with its role in quorum sensing, the spectrum of pigment and motility defects of a PA14 *vqsR* mutant paralleled those of *lasR* in our assays; the *vqsR* mutant was defective in both pyocyanin production and swarming, and displayed slightly reduced twitching (data not shown).
(TIF)

Figure S11 **Glutamate-cysteine ligase (** ***gshA*** **) is required for full virulence of PA14.** A) *gshA* (PA5203) is a single gene transcription unit. B) Four *MAR2xT7* transposon insertions in *gshA* are virulence-attenuated. Mutations in *gshA* and *gshB* (PA0407) exhibit a similar degree of attenuation suggesting that production of glutathione may be required for WT levels of virulence (see Figure 5D).
(TIF)

Figure S12 **Nine virulence-attenuated mutants exhibit normal growth.** Cultures were diluted from overnight saturated cultures to 10^4^ CFU/ml in M63 minimal media and grown at 37°C on a rotating wheel. At the indicated times, samples were taken of each culture, diluted and plated on LB plates. Plates were incubated overnight and colonies were counted the next day. A) Growth of virulence-attenuated *MAR2xT7* mutants *pchH* #23790, PA14_27700 #32578, PA2550 #34827, *pch*I #35711, PA0456 #36116, *clpA* #39351, PA1216 #47923, *vqsR* #52787. B) Growth of PA0745 in-frame deletion mutant.
(TIF)

Figure S13 **PA14 genes required for virulence in** ***C. elegans*** **were classified according to the breadth of their phylogenetic distribution across sequenced prokaryotes.** A) Phylogenetic tree indicating breadths of phylogenetic distribution (or phylostrata) from 0, the narrowest breadth corresponding to PA14-specific genes, and 1 corresponding to genes shared found only in other strains of *P. aeruginosa*, to 6, the root of the tree and the broadest breadth, corresponding to genes distributed across eubacteria and archea. Phylogenetic breadths were assigned to PA14 genes corresponding to the parent node uniting all the child taxa to which all orthologs of a particular gene are found. B) The percentages of *P. aeruginosa* strain PA14 genes, PA14-NR set genes, and primary, secondary, tertiary, auxotroph, and VFDB set genes within each phylogenetic breadth is shown. The primary, secondary, tertiary, and auxotroph gene sets were apparently underrepresented in the breadths 0, 1, 2, and 3, but without statistical significance after multiple comparison correction using false discovery rate (q< = 0.05) due to the small number of genes involved. Among the auxotrophs, genes of breadth 4 were underrepresented (p-value = 0.0027) and 6 overrepresented (p-value = 1.2×10^−8^). Among VFDB genes, genes of breadth 3 were overrepresented (p-value = 1.22×10^−8^) and breadth 6 were underrepresented (p-value = 7.92×10^−11^).
(TIF)

Table S1 **Numerical accounting of virulence-attenuated PA14 transposon mutants identified in the primary, secondary and tertiary screens.** (*) The 5754 mutants screened was the NR library (5850 insertion mutants) minus one 96-well plate of known slow growing mutants. Note that for the primary and secondary screen, the number of mutants obtained is presented; some genes were represented by multiple mutants and a few mutants were in intergenic regions (see text). In the tertiary screen, the number of genes is designated.
(DOCX)

Table S2 **Auxotrophic and slow-growing mutants identified in the primary screen for virulence-attenuated mutants.**
(XLSX)

Table S3 **Virulence-attenuated mutants identified in the primary screen minus auxotrophs from Table S2.**
(XLSX)

Table S4 **PA14 virulence-attenuated mutants identified in the secondary screen.**
(XLSX)

Table S5 **Mutants in 58 genes tested in the tertiary screen for attenuation of virulence.**
(XLSX)

Table S6 **41 virulence-attenuated genes shown in** **Figure 2** **.**
(XLSX)

Table S7 **Contains the following sheets showing totals and statistics for** **Figures 7** **,** **8** **, and S13.** This spreadsheet contains total counts, and percentages for comparisons across the various gene sets: the PA14 genome, the NR set, and the primary, secondary, tertiary, auxotroph, and VFDB sets. Fisher exact tests were performed relative to gene totals the NR set, and left, right, and two-tailed probabilities (p-values) indicating underrepresentation, overrepresentation, or extreme value, respectively, are shown for the primary, secondary, tertiary, auxotroph, and VFDB sets. FDR q-values are calculated only for the left and right probabilities, and are shown below the corresponding left and right probabilities. Results with statistical significance after FDR correction are shown in red font. Cells are colored according to the coloring of the corresponding legends in Figure 7 and Figure 8. Sheet “A. Core vs Auxiliary Genes”. This sheet contains the total counts and percentages of Core and Auxiliary genes across the various gene sets (see above). These data are presented graphically in Figure 7A with corresponding coloring of bars. Sheet “B. Predicted vs Non-Island”. This sheet contains the total counts and percentages of “predicted island” and “non-island” genes across the various gene sets. These data are presented graphically in Figure 7B. Sheet “C. Known Island vs Non-Island”. This sheet contains the total counts and percentages of “known island” and “non-island” genes across the various gene sets. These data are presented graphically in Figure 7C. Sheet “D. Phylogenetic Breadths”. This sheet contains the total counts and percentages of genes with various breadths of phylogenetic distribution, or phylostrata across the various gene sets. These data are presented graphically in Figure S13. Sheet “E. PGS and HFBP”. This sheet contains the total counts and percentages of genes within the *Pseudomonas*-genus-specific (PGS), in the HFBP set, and in non-PGS non-HFBP (“All Others”), across the various gene sets. These data are presented graphically in Figure 8.
(XLS)
