# Supplementary figures and images for: Genome-Wide Identification of Pseudomonas aeruginosa Virulence-Related Genes Using a Caenorhabditis elegans Infection Model
Source: PLoS Pathog. 2012 Jul 26;8(7):e1002813. doi: 10.1371/journal.ppat.1002813 (PMC3406104; doi:10.1371/journal.ppat.1002813)

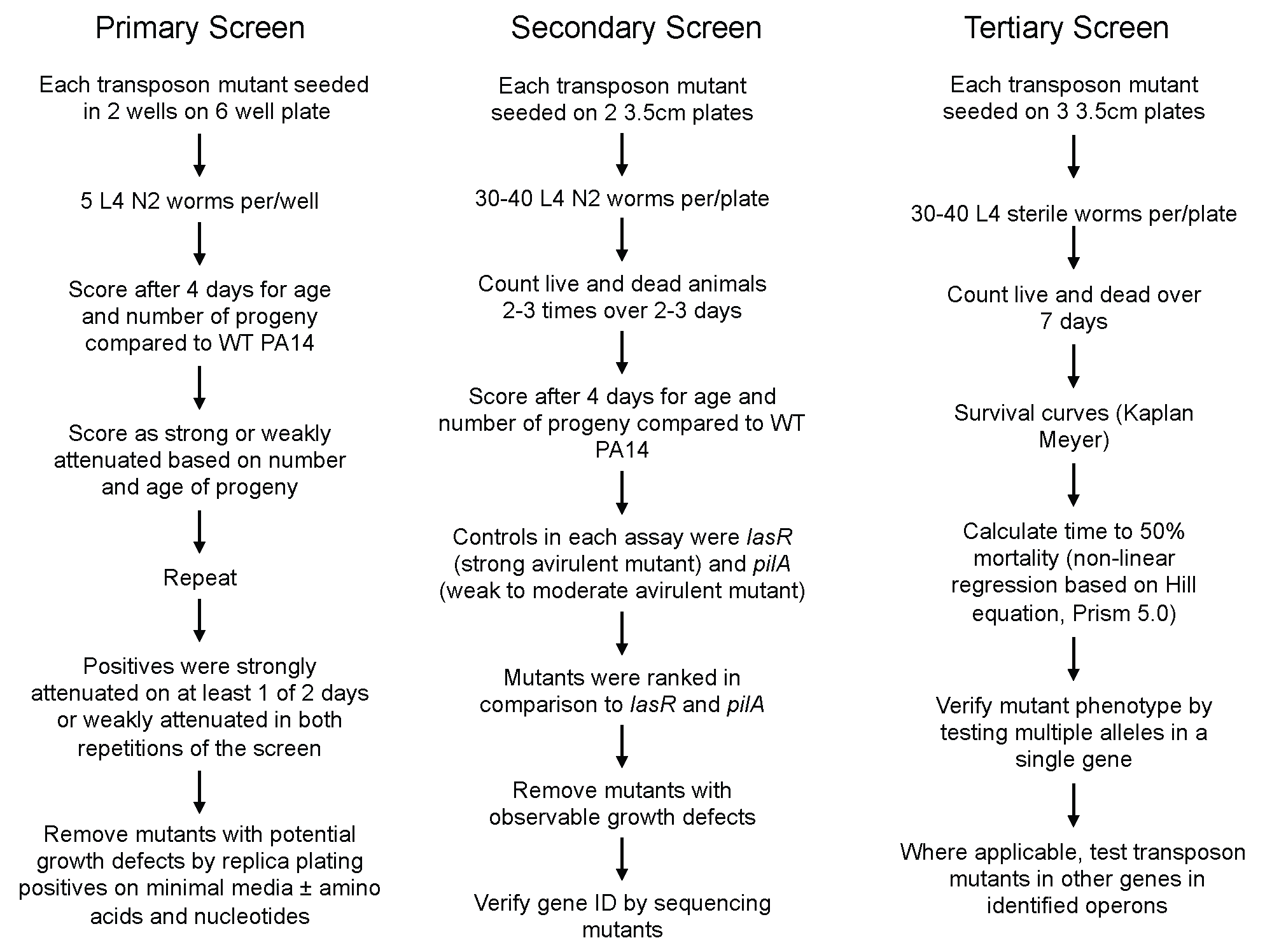

Supplement: Figure S1 — Flow chart of screening procedure for virulence-attenuated PA14 transposon mutants in C. elegans . (TIF) [file ppat.1002813.s001.tif]

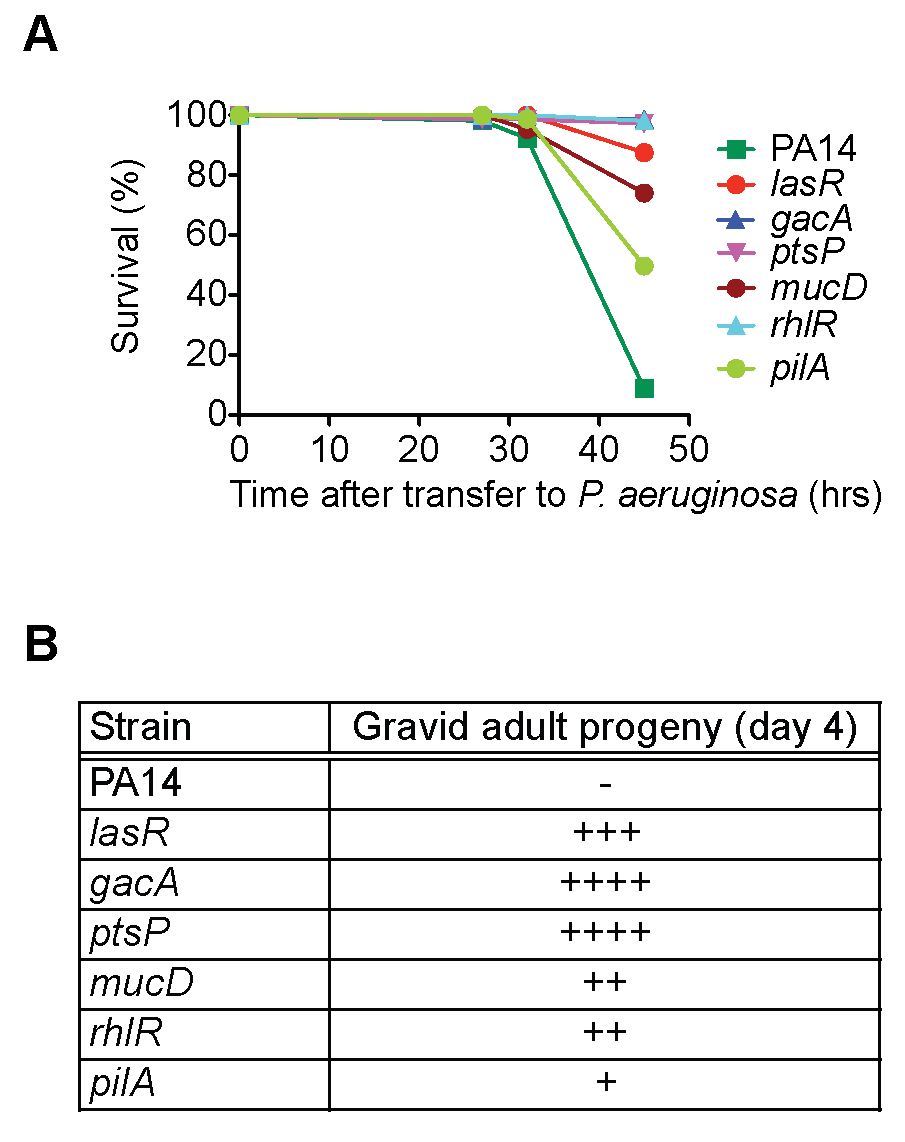

Supplement: Figure S2 — Correlation of virulence attenuation in standard SK and progeny survival assays. A) lasR, gacA, ptsP, mucD, rhlR and pilA mutants all have attenuated virulence in a standard slow killing (SK) assay (60–80 N2 worms tested). B) The number and developmental stage of the progeny on the SK plates in panel (A) four days after transfer of the parent worms to pathogen at 25°C. On PA14, there were few progeny and no gravid adults were observed whereas gravid adult progeny were found on the virulence-attenuated P. aeruginosa strains. The number and age of progeny on the mutant plates was qualitatively scored in comparison to the PA14 WT plates. Plates seeded with gacA and ptsP were overrun with hundreds of gravid adult worms that consumed the bacterial lawn. lasR, mucD, rhlR and pilA plates contained fewer gravid adult worms but all were observably greater than plates seeded with WT PA14. (TIF) [file ppat.1002813.s002.tif]

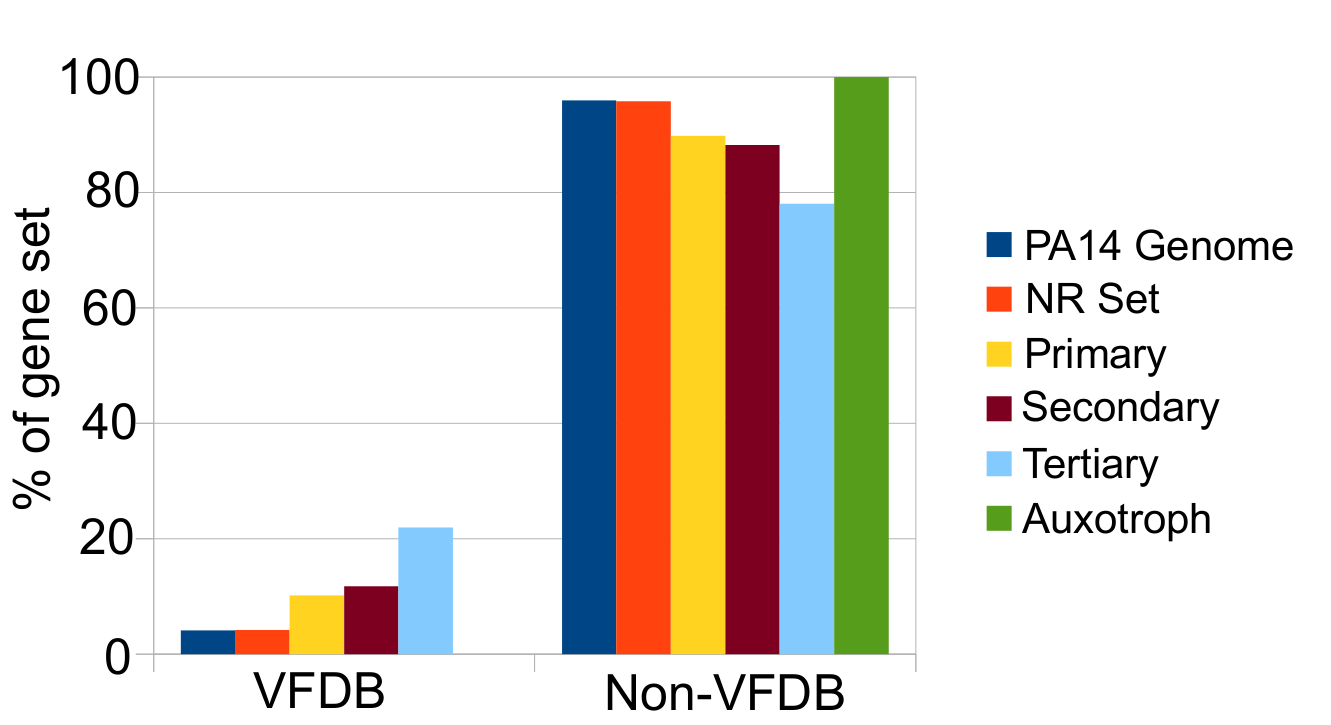

Supplement: Figure S3 — Percentage of mutants that are VFDB genes. The percentage of primary, secondary, and tertiary, and auxotroph set virulence-attenuated genes that are VFDB and non-VFDB genes are indicated. VFDB genes are significantly overrepresented in the primary, secondary, and tertiary sets, with p-values of 5.5×10^−6^, 2.1×10^−5^, and 3.7×10^−5^, respectively. Furthermore, their overrepresentation increases with successive screen iterations. (TIF) [file ppat.1002813.s003.tif]

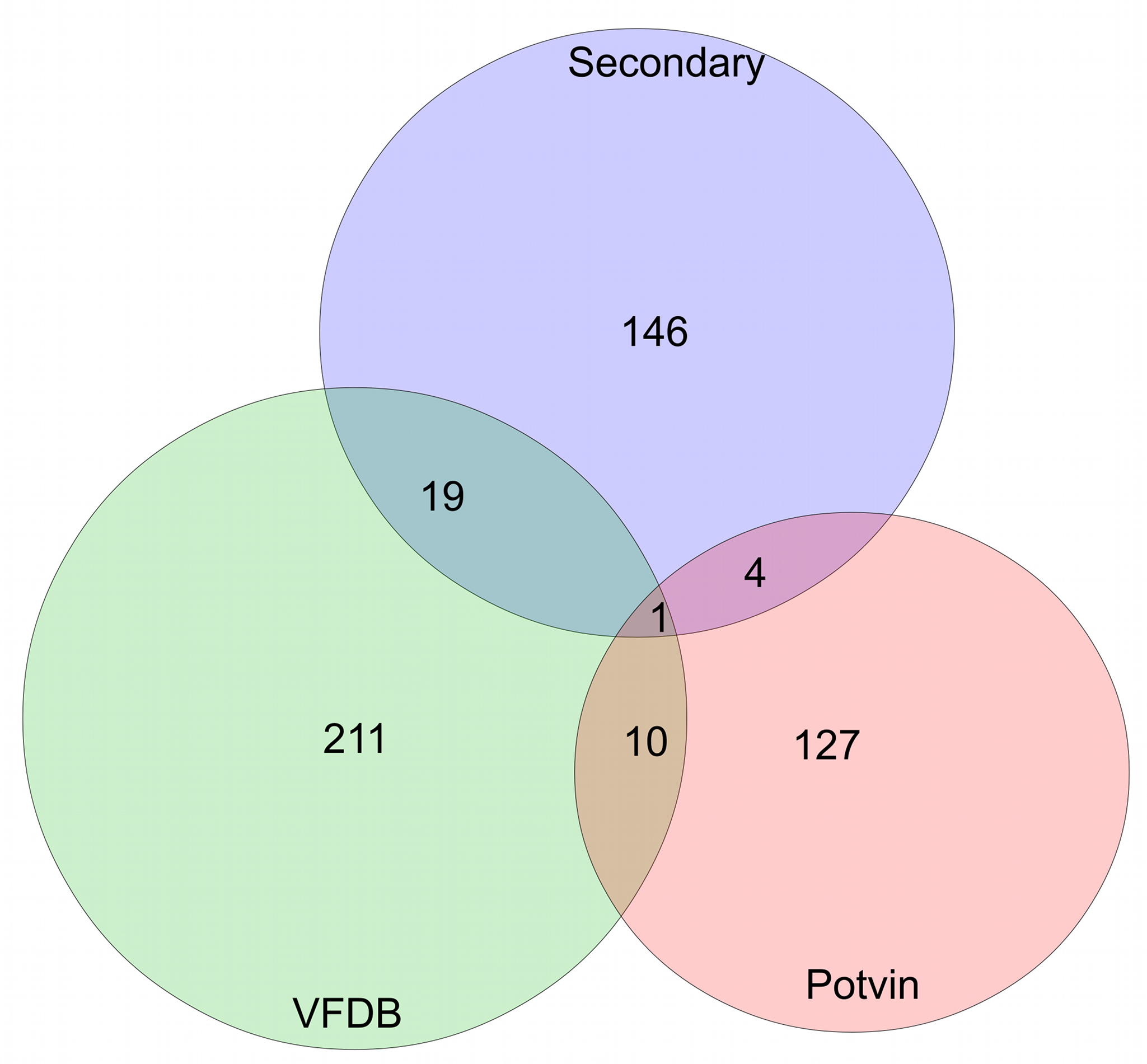

Supplement: Figure S4 — Venn diagram showing the overlaps between the 170 virulence-attenuated genes obtained in the secondary screen, the Potvin set, and the PA14 VFDB set. (TIF) [file ppat.1002813.s004.tif]

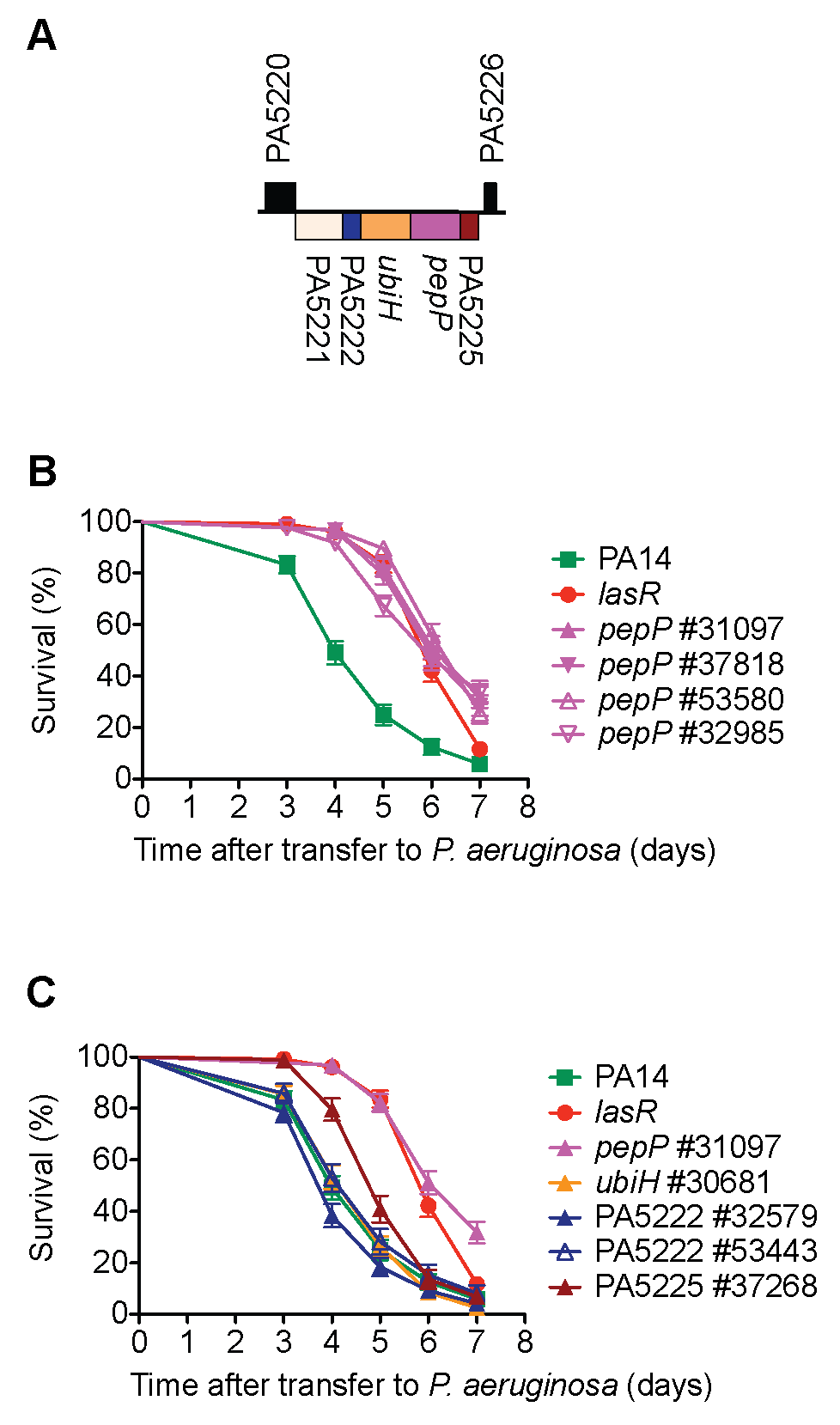

Supplement: Figure S5 — Multiple pepP alleles but not alleles of other genes in the operon have attenuated virulence. A) PepP is a cytoplasmic aminopeptidase that cleaves aminoacyl proline dipeptides from the N terminus of polypeptides. In PA14 pepP appears to be the second gene in a 5 gene operon comprised of PA5225, a hypothetical protein, pepP, ubiH a ubiquinone biosynthetic enzyme, PA5225 another hypothetical protein, and terminating with PA5221, an ORF with homology to E. coli visC a pyridine nucleotide-disulphide oxidoreductase in the ubiH family. B) Four MAR2xT7 alleles of pepP display highly attenuated C. elegans killing. C) Transposon mutants in 4/5 genes in the pepP operon were tested for their effect on virulence (no mutant was available in PA5221). Mutants in the two downstream genes tested, ubiH and PA5222 exhibited wild-type levels of virulence whereas the single mutant in the upstream PA5225 had a modest attenuation of virulence (compared to pepP) that might be due to effects on pepP expression. pepP mutant #31097 produced elevated levels of pyocyanin and had somewhat reduced swimming ability, although swarming was normal on both SK and LB (Table 2). The PepP proline aminopeptidase could function in utilization of exogenous peptides as nutrients, degradation of proteins or protein maturation, perhaps of a specific substrate relevant to virulence. There is a precedent for aminopeptidase function being linked to virulence associated phenotypes. P. aeruginosa PepA was shown to be involved in the regulation of alginate biosynthesis [100]. (TIF) [file ppat.1002813.s005.tif]

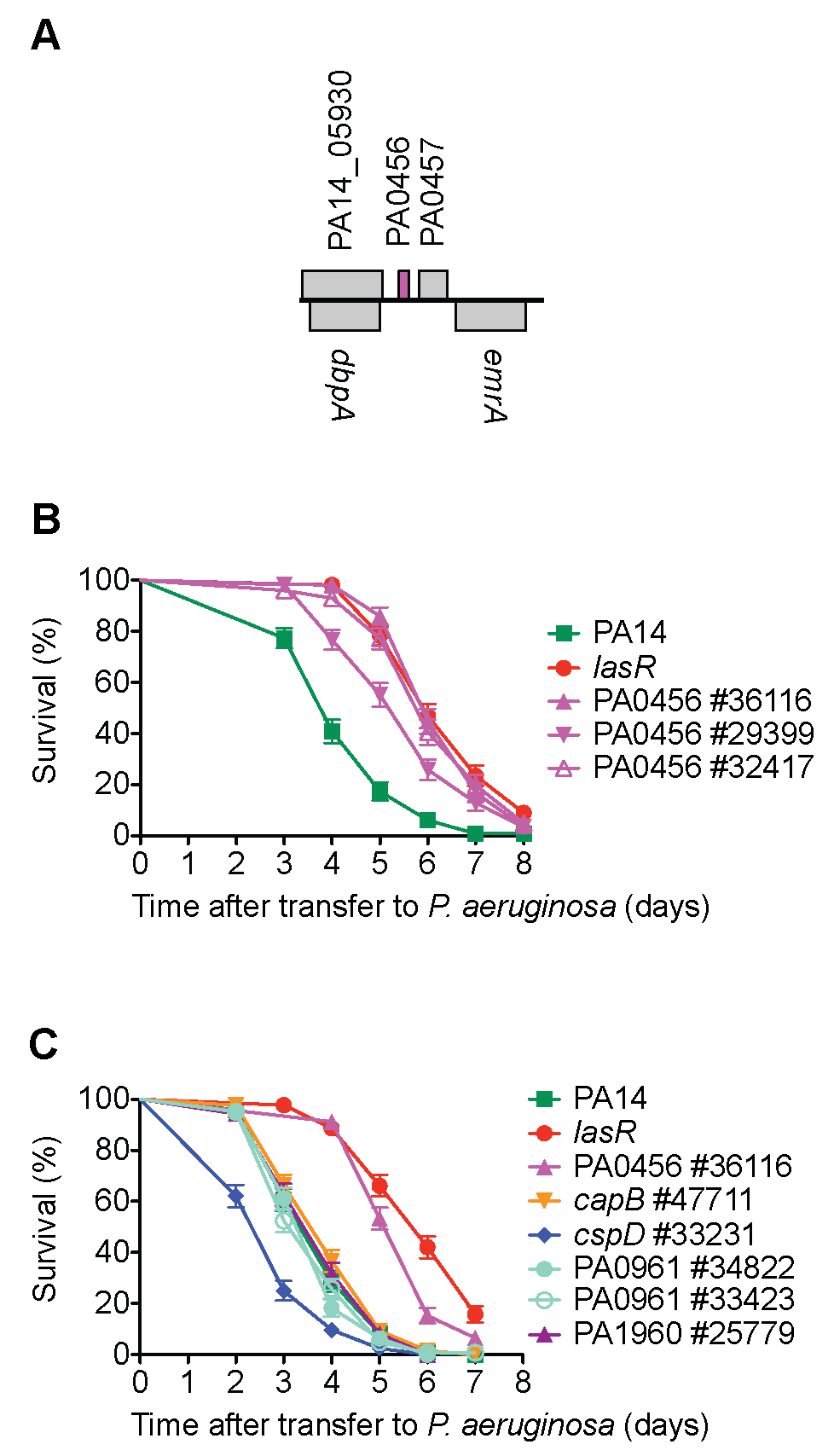

Supplement: Figure S6 — Mutations in cold shock domain (CSD) protein PA0456 are virulence-attenuated in C. elegans but mutations in four other CSD proteins do not affect virulence. A) PA0456 is most likely a single gene transcription unit. PA0456 (previously annotated as cspB in the PA14 genome) contains a canonical cold shock domain (CSD) and is homologous to other CSD proteins (57% identical to the major E. coli cold shock protein, CspA, and 65% to B. subtilis CspB as determined by BLASTP). Although the first cold shock proteins (Csps), a conserved family of small mostly acidic proteins that bind single stranded DNA and RNA, were identified as major proteins induced upon temperature downshift, some members of the family are not induced upon cold shock and many are implicated in other cellular functions [101]. B) Three independent MAR2xT7 transposon insertions in PA0456 have attenuated virulence in C. elegans. C) Transposon insertion mutants in four additional CSD containing genes (capB PA3266, PA0961, cspD PA2622 and PA1960) have wild-type virulence in C. elegans. Five additional CSD-containing homologs of PA0456 (PA3266, PA1159, PA0961, PA2622, PA1960) were identified by BLASTP against the PA14 protein database with 76, 66, 62, 54 and 41% identity to PA0456 respectively; all 5 contain a CSD as analyzed by Prosite and transposon insertion mutants were available in 4/5 of these genes. Among the CSD containing proteins tested, PA0456 appears to be unique in its role in virulence, suggesting that the cold shock response per se is most likely not required for virulence of PA14. PA14 PA0456 has been reported to be regulated by quorum sensing [102], and in keeping with these findings PA0456 exhibited defects in quorum sensing regulated phenotypes; the PA0456 mutant had reduced pyocyanin production and swarming motility (Table 2). (TIF) [file ppat.1002813.s006.tif]

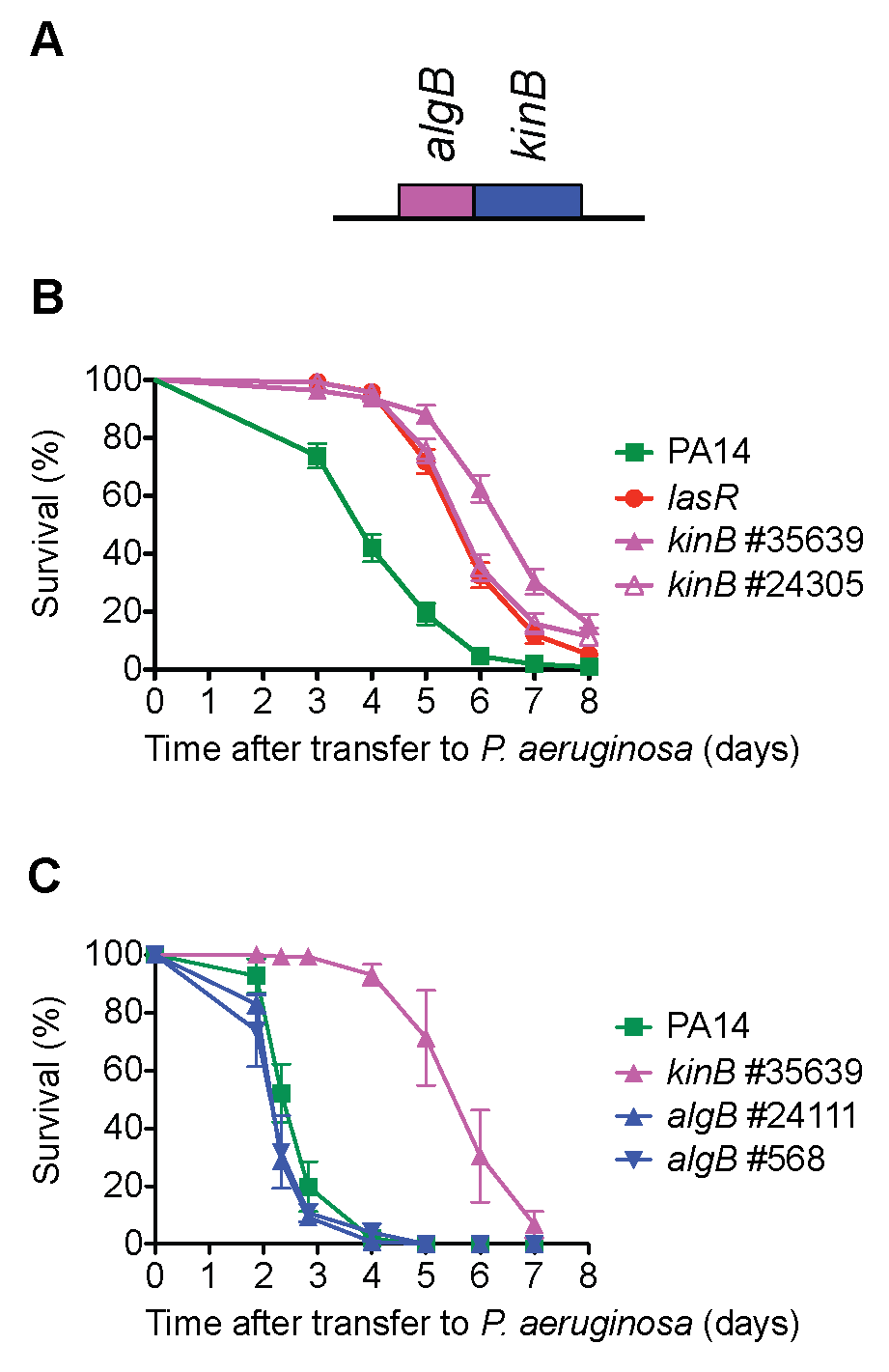

Supplement: Figure S7 — KinB (PA5484) sensor kinase is required for PA14 virulence in C. elegans . KinB negatively regulates alginate production and has been recently shown to be required for virulence in zebrafish embryos and mice [86], [103]. A) kinB and its cognate response regulator algB form a two gene operon. B) Two MAR2xT7 transposon alleles in kinB (as well as an in-frame deletion of kinB described in Chand et al., data not shown) are reduced in virulence. C) Transposon mutants in algB (and an in-frame deletion of algB described in Chand el al., data not shown) exhibited wild-type virulence in C. elegans, paralleling their lack of phenotype in zebrafish [86]. (TIF) [file ppat.1002813.s007.tif]

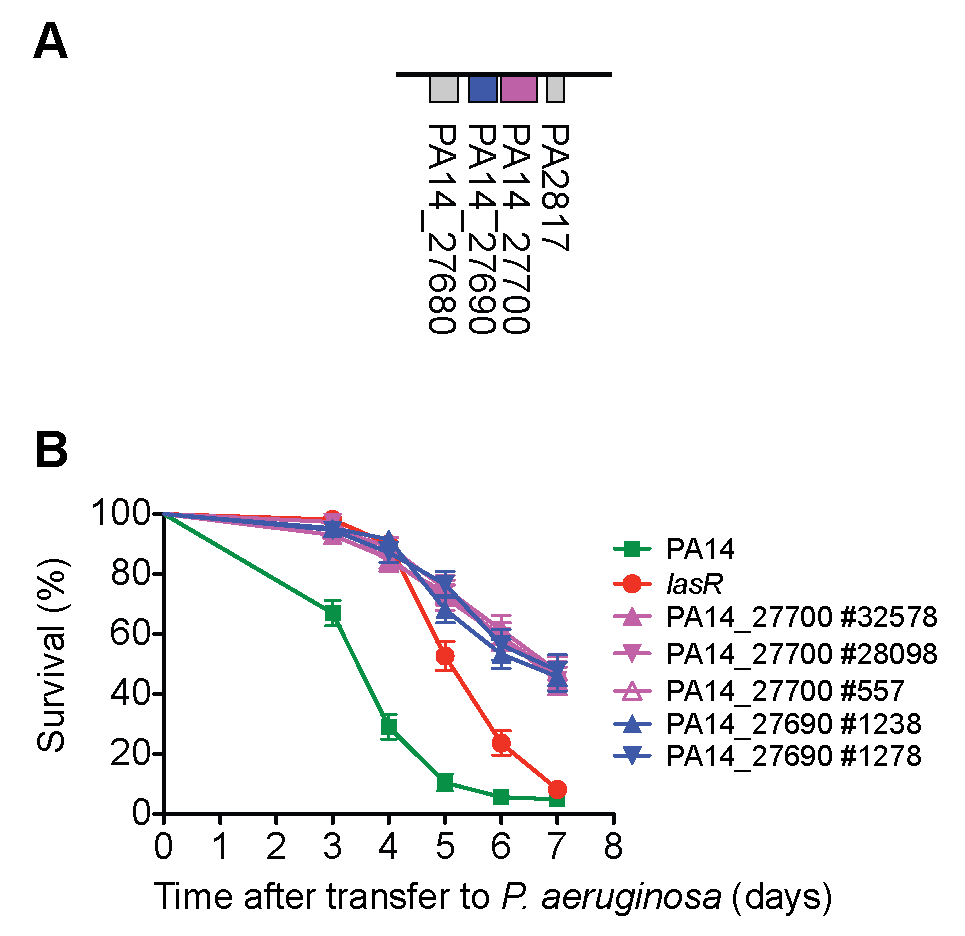

Supplement: Figure S8 — Mutations in putative transcriptional regulator PA14_27700 and possible ECF sigma factor PA14_27690 are virulence-attenuated in C. elegans . A) PA14_27700, a Crp/FNR-type transcriptional regulator that appears to have no homologue in PA01 [32], is located 264 bp downstream of PA2817 and 66 upstream of PA14_26790, a FecI-like extracytoplasmic function (ECF) sigma that also has no PA01 homologue. It is unclear whether PA14_27700 and PA14_26790 form an operon. B) Multiple MAR2xT7 insertions in both PA14_27700 and PA14_27690 are attenuated in virulence. Although not initially identified in our screen, mutants in PA14_26790 have a strong virulence-attenuated phenotype like PA14_27700. ECF sigma factors, to which PA14_26790 has homology, are commonly co-transcribed with a regulatory anti-sigma factor and the ECF sigma factor and its regulatory anti-sigma factor frequently play an important role in adaptation to the external environment [104]. Canonical transmembrane anti-sigma factors have a small cytoplasmic regulatory/inhibitory domain linked by a transmembrane domain to a C-terminus sensory domain that resides in the periplasm. PA14_27700 has neither a signal sequence nor a transmembrane domain suggesting that it functions in the cytoplasm. However, most anti-sigma factors are poorly conserved at the sequence level and there are anti-sigma factors that sense cytoplasmic stimuli. PA14_27700 #32578, although attenuated in virulence, does not exhibit any obvious defects in pigment production or motility in our assays (Table 2). (TIF) [file ppat.1002813.s008.tif]

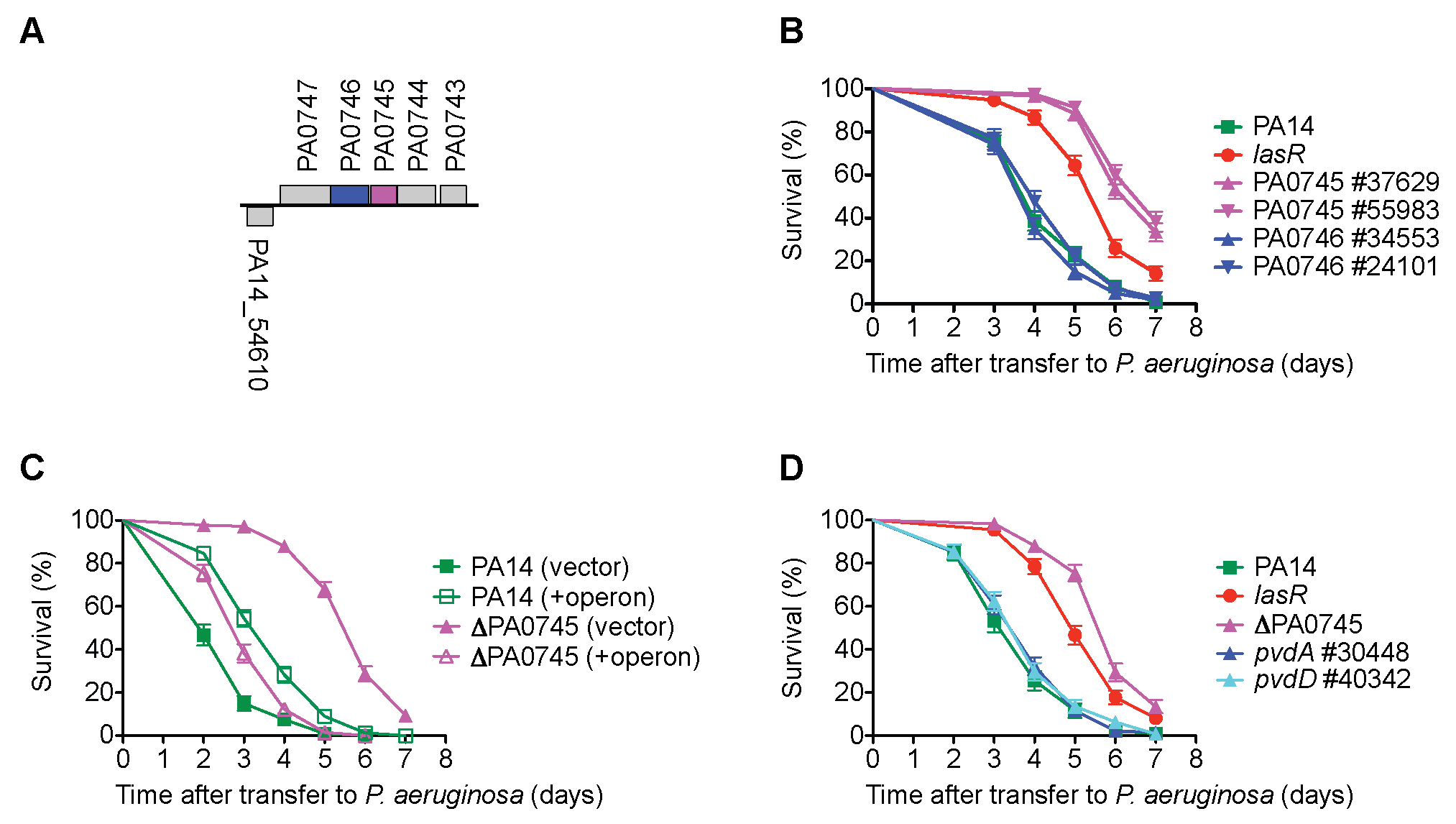

Supplement: Figure S9 — The virulence-attenuated phenotype of MAR2xT7 insertions in putative enoyl-CoA hydratase isomerase PA0745 is recapitulated by an in-frame deletion and is not dependent on pyoverdine production. A) PA0745, a putative enoyl-CoA hydratase isomerase, is the third gene of what is most likely a 5 gene operon: PA0747 a probable aldehyde dehydrogenase, PA0746 a putative acyl-CoA dehydrogenase, PA0745, PA0744 another putative enoyl-CoA hydratase/isomerase, and PA0743 a probable 3-hydroxyisobutyrate dehydrogenase. B) Insertions in PA0745 exhibit a virulence-attenuated phenotype but mutants of PA0746, the only other gene in the putative operon for which MAR2xT7 mutants were available, exhibited wild-type levels of virulence. C) The virulence-attenuated phenotype of an in-frame deletion mutant of PA0745 is complemented by expression of the entire operon (PA0747-PA0743) in trans. The operon was expressed under its own promoter; PA14 DNA from genome position 4851671–4848075 was cloned in Pseudomonas vector pucP19 [105]. D) A defect in pyoverdine production in PA0745 mutants (Table 2) is not the cause of the PA0745 avirulent phenotype. Pyoverdine biosynthetic mutants pvdA and pvdD show minimal to no attenuation of virulence. It has been suggested that PA0745 enoyl-CoA hydratase isomerase may function in the production of cis-2-decenoic acid, a fatty acid signal responsible for inducing biofilm dispersal, due to its weak homology to RpfF which produces cis-11-methyl-2-dodecenoic acid, the diffusible soluble factor (DSF) required for virulence in Xanthomonas campestris [106], [107]. However, PA0745 and X. campestris paaF (required for the breakdown of phenlyacetic acid) are reciprocal top BLASTP hits with 65% identity over 95% of the protein. The putative enzymatic functions of the genes in the PA0745 operon suggests that this operon may collectively synthesize or degrade an as yet unidentified product/products that affect siderophore production, control of swarming, and virulence i [file ppat.1002813.s009.tif]

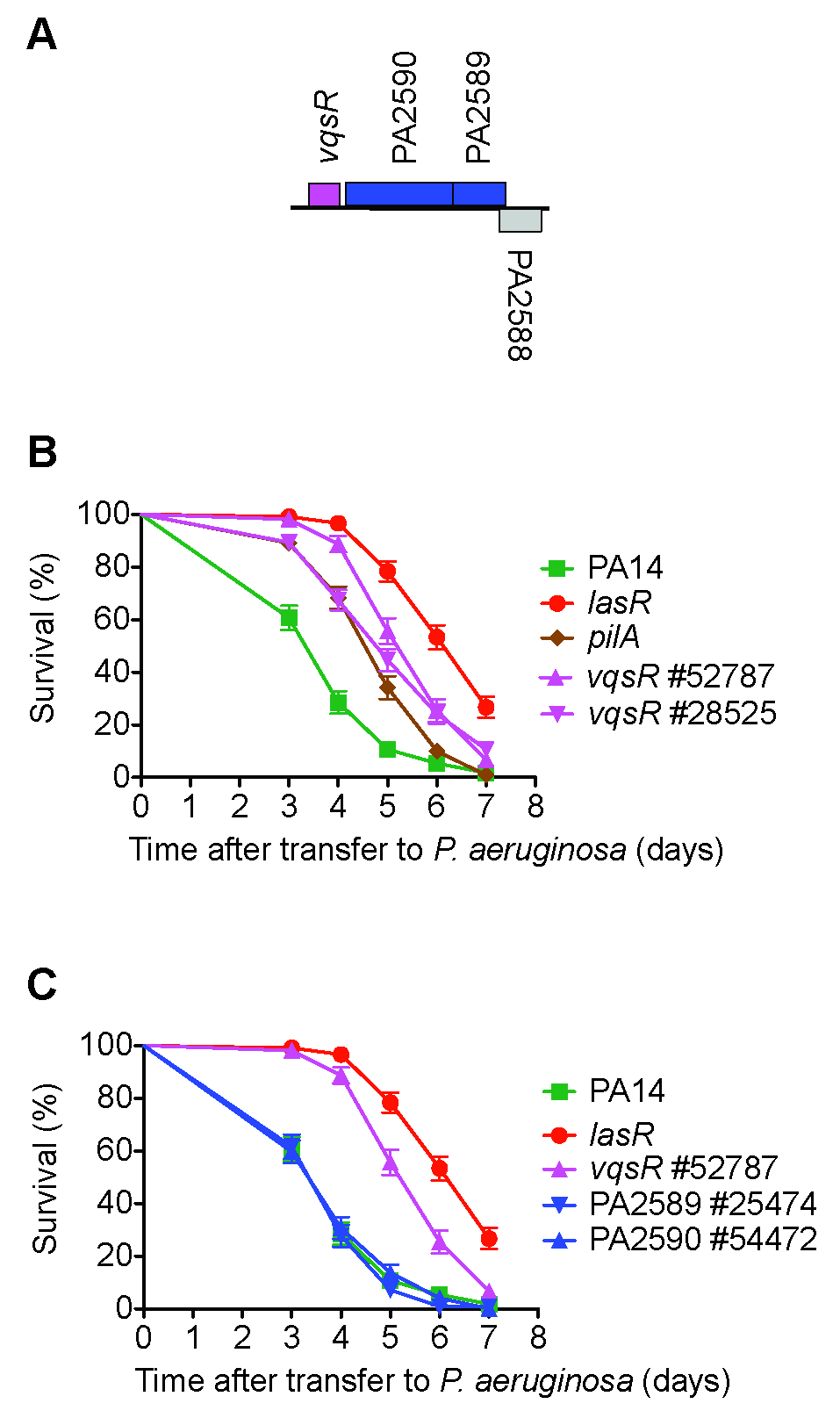

Supplement: Figure S10 — Transposon mutants in transcriptional regulator vqsR , but not genes in the adjacent downstream operon, are attenuated in virulence. VqsR (PA2591) is a LuxR type transcriptional regulator that controls expression of quorum sensing and virulence genes. A vqsR mutant of P. aeruginosa strain TB has been shown to be impaired in homoserine lactone production and is attenuated in a liquid C. elegans killing assay that occurs over a period of hours, similar in kinetics to toxin mediated PA14 “fast-killing” [108]. A) P. aeruginosa PA14 vqsR appears to be transcribed as an individual ORF; it is located 120 bp upstream of an operon that encodes two conserved hypothetical proteins, PA2590 a putative outer membrane receptor protein and PA2589 a possible permease. B) Two MAR2xT7 vqsR transposon mutants have reduced virulence in C. elegans. C) MAR2xT7 mutants in the operon adjacent to and downstream of vqsR (PA2590 and PA2589) are as virulent as WT PA14. In keeping with its role in quorum sensing, the spectrum of pigment and motility defects of a PA14 vqsR mutant paralleled those of lasR in our assays; the vqsR mutant was defective in both pyocyanin production and swarming, and displayed slightly reduced twitching (data not shown). (TIF) [file ppat.1002813.s010.tif]

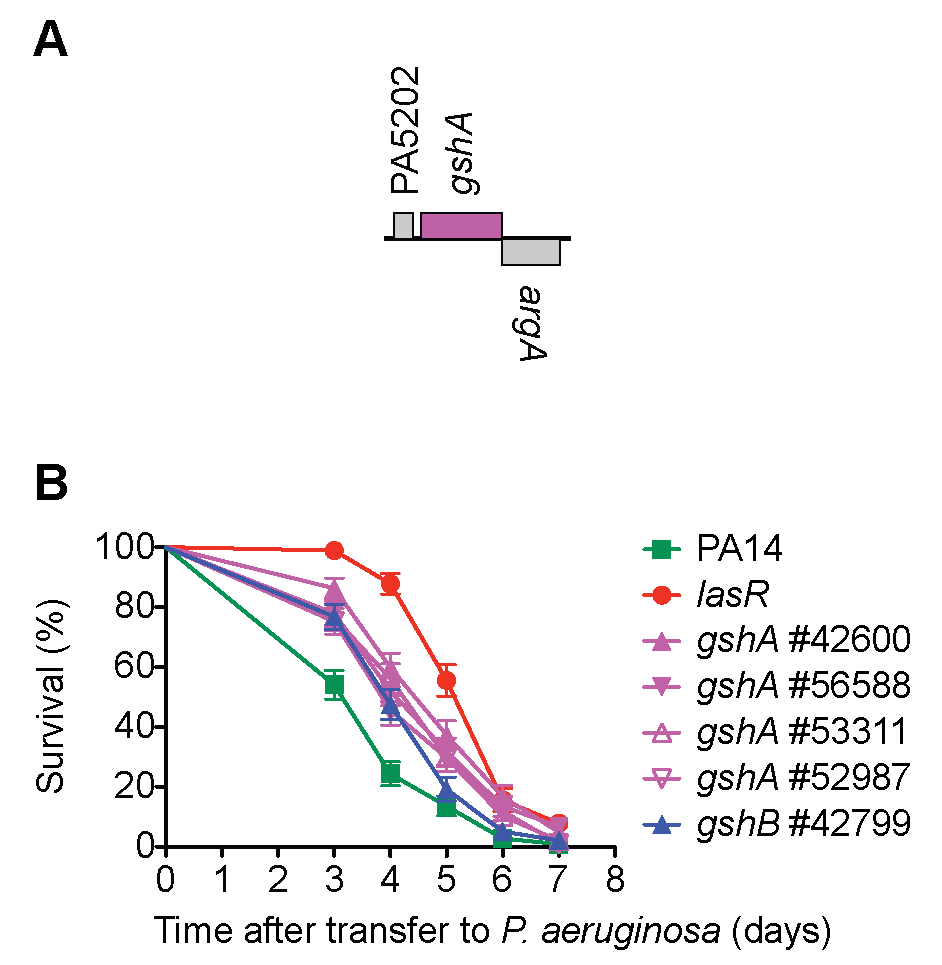

Supplement: Figure S11 — Glutamate-cysteine ligase ( gshA ) is required for full virulence of PA14. A) gshA (PA5203) is a single gene transcription unit. B) Four MAR2xT7 transposon insertions in gshA are virulence-attenuated. Mutations in gshA and gshB (PA0407) exhibit a similar degree of attenuation suggesting that production of glutathione may be required for WT levels of virulence (see Figure 5D). (TIF) [file ppat.1002813.s011.tif]

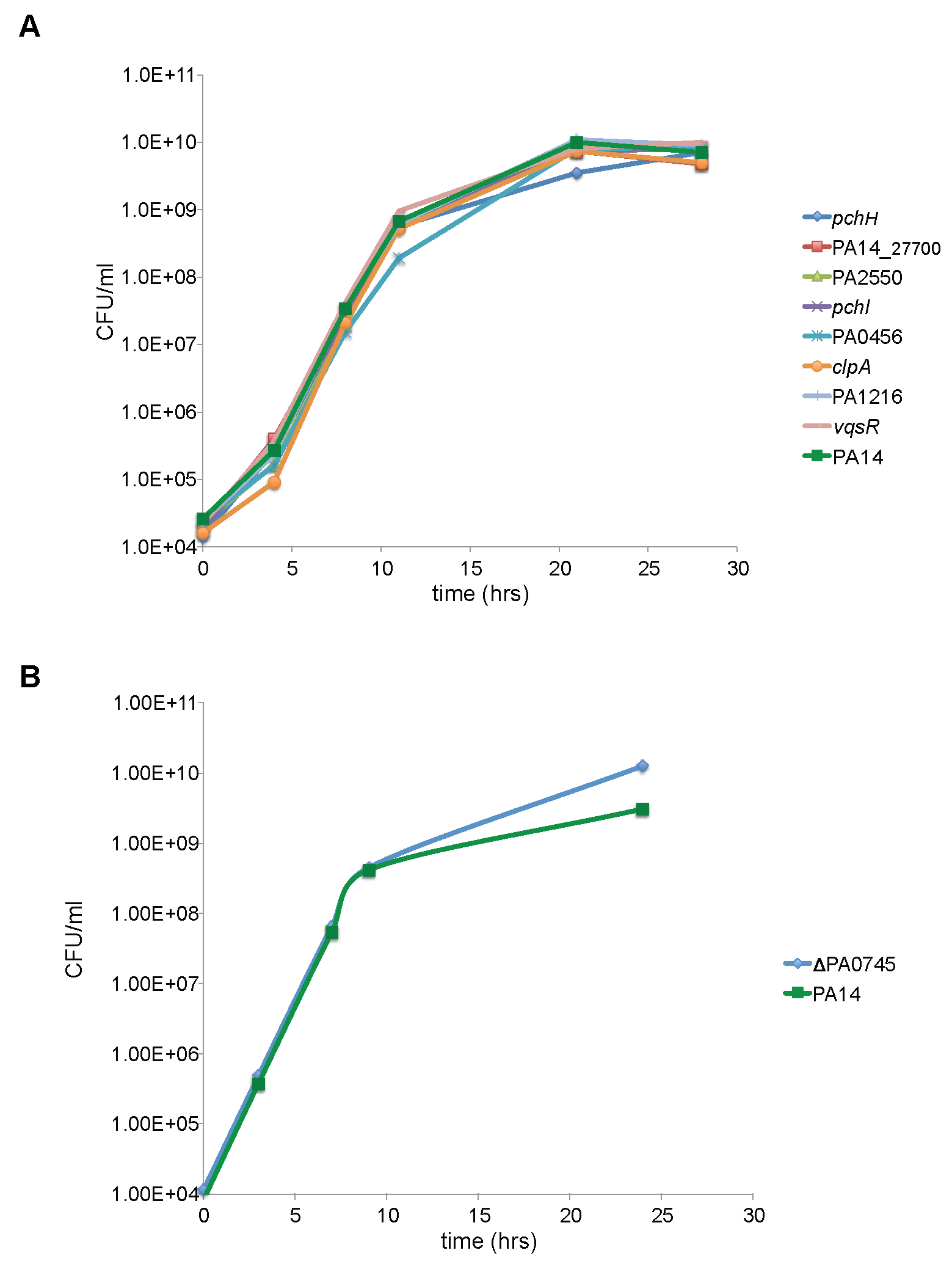

Supplement: Figure S12 — Nine virulence-attenuated mutants exhibit normal growth. Cultures were diluted from overnight saturated cultures to 104 CFU/ml in M63 minimal media and grown at 37°C on a rotating wheel. At the indicated times, samples were taken of each culture, diluted and plated on LB plates. Plates were incubated overnight and colonies were counted the next day. A) Growth of virulence-attenuated MAR2xT7 mutants pchH #23790, PA14_27700 #32578, PA2550 #34827, pchI #35711, PA0456 #36116, clpA #39351, PA1216 #47923, vqsR #52787. B) Growth of PA0745 in-frame deletion mutant. (TIF) [file ppat.1002813.s012.tif]

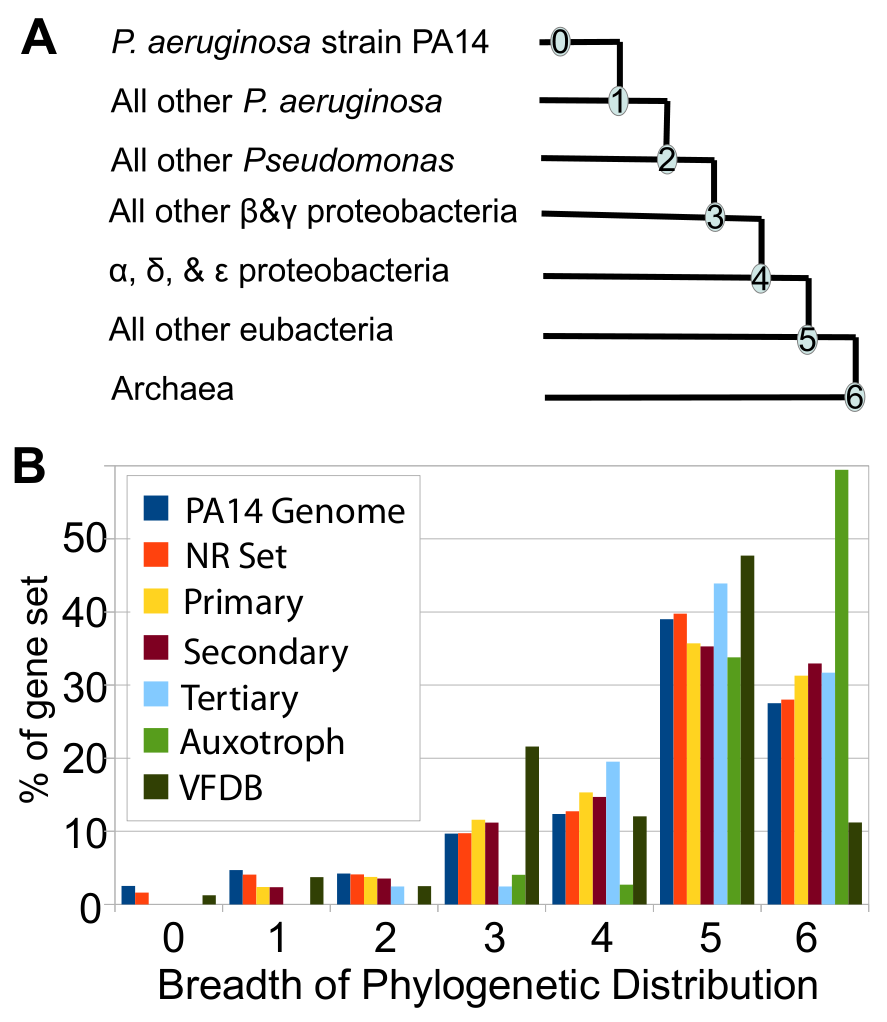

Supplement: Figure S13 — PA14 genes required for virulence in C. elegans were classified according to the breadth of their phylogenetic distribution across sequenced prokaryotes. A) Phylogenetic tree indicating breadths of phylogenetic distribution (or phylostrata) from 0, the narrowest breadth corresponding to PA14-specific genes, and 1 corresponding to genes shared found only in other strains of P. aeruginosa, to 6, the root of the tree and the broadest breadth, corresponding to genes distributed across eubacteria and archea. Phylogenetic breadths were assigned to PA14 genes corresponding to the parent node uniting all the child taxa to which all orthologs of a particular gene are found. B) The percentages of P. aeruginosa strain PA14 genes, PA14-NR set genes, and primary, secondary, tertiary, auxotroph, and VFDB set genes within each phylogenetic breadth is shown. The primary, secondary, tertiary, and auxotroph gene sets were apparently underrepresented in the breadths 0, 1, 2, and 3, but without statistical significance after multiple comparison correction using false discovery rate (q< = 0.05) due to the small number of genes involved. Among the auxotrophs, genes of breadth 4 were underrepresented (p-value = 0.0027) and 6 overrepresented (p-value = 1.2×10^−8^). Among VFDB genes, genes of breadth 3 were overrepresented (p-value = 1.22×10^−8^) and breadth 6 were underrepresented (p-value = 7.92×10^−11^). (TIF) [file ppat.1002813.s013.tif]
