# Supplementary material for: Genome-Wide Identification of Pseudomonas aeruginosa Virulence-Related Genes Using a Caenorhabditis elegans Infection Model
Source: PLoS Pathog. 2012 Jul 26;8(7):e1002813. doi: 10.1371/journal.ppat.1002813 (PMC3406104; doi:10.1371/journal.ppat.1002813)
Supplement: Supplementary file 2 [file ppat.1002813.s014.docx]

	Progeny Screen	SK in N2	SK in *fer-15;fem-1*
	Primary (# mutants)	Secondary (# mutants)	Teritary (# genes)
Number screened	*5754	313	58
Initial positives	399	198	
Auxotrophs/slow growers replica plating on minimal	86	ND	
Rough colony variants		4	
Observable growth defect in LB or M63		5	
Sequence incorrect		9	
Sequence not determined		[12]	
Did not repeat in *fer-15;fem-1* tertiary screen			4
Did not repeat with multiple alleles			8
Growth problems			3
Other			2
TOTAL	313	180	41
Strong = *lasR*		34	25
Moderate ≥ *pilA*		76	16
Weak > PA14 or progeny only		70	
